# Depletion of carbohydrate reserves limits nitrate uptake during early regrowth in *Lolium perenne* L.

**DOI:** 10.1093/jxb/erx056

**Published:** 2017-03-31

**Authors:** Qianqian Guo, Matthew Hamish Turnbull, Jiancheng Song, Jessica Roche, Ondrej Novak, Jana Späth, Paula Elizabeth Jameson, Jonathan Love

**Affiliations:** 1 School of Biological Sciences, University of Canterbury, Private Bag 4800, Christchurch, New Zealand; 2 School of Life Sciences, Yantai University, Yantai 264005, China; 3 Laboratory of Growth Regulators, Centre of the Region Haná for Biotechnological and Agricultural Research, Institute of Experimental Botany CAS & Faculty of Science of Palacký University, Šlechtitelů 27, 783 71 Olomouc, Czech Republic; 4 Swedish Metabolomics Centre, Department of Forest Genetics and Plant Physiology, Swedish University of Agricultural Sciences KBC, Umeå University, Linnéus väg 6, SE-90183 Umeå, Sweden

**Keywords:** Carbohydrate, carbon, cytokinin, fructan, *Lolium perenne*, nitrate transporter (NRT), nitrate uptake, nitrogen, nitrogen use efficiency (NUE), perennial ryegrass.

## Abstract

The mechanisms linking C/N balance to N uptake and assimilation are central to plant responses to changing soil nutrient levels. Defoliation and subsequent regrowth of grasses both impact C partitioning, thereby creating a significant point of interaction with soil N availability. Using defoliation as an experimental treatment, we investigated the dynamic relationships between plant carbohydrate status and NO_3_^–^-responsive uptake systems, transporter gene expression, and nitrate assimilation in *Lolium perenne* L. High- and low-affinity NO_3_^–^ uptake was reduced in an N-dependent manner in response to a rapid and large shift in carbohydrate remobilization triggered by defoliation. This reduction in NO_3_^–^ uptake was rescued by an exogenous glucose supplement, confirming the carbohydrate dependence of NO_3_^–^ uptake. The regulation of NO_3_^–^ uptake in response to the perturbation of the plant C/N ratio was associated with changes in expression of putative high- and low-affinity NO_3_^–^ transporters. Furthermore, NO_3_^–^ assimilation appears to be regulated by the C–N status of the plant, implying a mechanism that signals the availability of C metabolites for NO_3_^–^ uptake and assimilation at the whole-plant level. We also show that cytokinins may be involved in the regulation of N acquisition and assimilation in response to the changing plant C/N ratio.

## Highlight

A shift in C storage triggered by defoliation has a significant impact on nitrate uptake in an N-status-dependent manner. Cytokinins are possible co-ordinators of C and N metabolism and N acquisition at the whole-plant level.

## Introduction

Grasses are well adapted to tolerate and recover from the severe and frequent defoliation associated with grazing (Lestienne *et al.*, 2006). The most obvious developmental aspect of this is the physical protection of the meristem at the crown. Less overt, but no less critical, are the metabolic aspects of this tolerance. For instance, leaf regrowth and the re-establishment of photosynthesis in the leaf prior to its transition from sink to source is dependent upon the remobilization of stored nutrient resources, particularly carbon (C) and nitrogen (N) (Ourry *et al.*, 1989; Lestienne *et al.*, 2006). In the period immediately following defoliation, photosynthetic capacity is temporarily compromised. This interrupts C assimilation and the capacity to assimilate newly acquired inorganic N into biomass, which is a process requiring reducing equivalents, ATP, and C skeletons generated from respiration of sucrose derived from stored C (Dawar *et al.*, 2010; Nunes-Nesi *et al.*, 2010). Indeed, it has been established that root growth, inorganic N uptake, respiration, and nitrate assimilation decline rapidly after defoliation (Boucaud and Bigot, 1989; Ourry *et al.*, 1989; Richards, 1993; Louahlia *et al.*, 2008).

Some evidence exists to suggest that C and N metabolism is co-ordinated via a sensing of plant C/N balance, and regulated by the expression of genes involved in photosynthesis, metabolic pathways, protein degradation, and N assimilation (Stitt and Krapp, 1999; Coruzzi and Zhou, 2001; Palenchar *et al.*, 2004; Gutiérrez *et al.*, 2007). Microarray data from Arabidopsis transiently exposed to a matrix of C and N treatments has indicated that a large proportion of genes respond to a C/N interaction, suggesting C and/or N, or a metabolic product of C and N assimilation (e.g. an amino acid), might act as a signal in the regulation of gene expression (Gutiérrez *et al.*, 2007). Despite several studies proposing possible mechanisms underlying C and N signalling in Arabidopsis, a definitive mechanistic understanding remains elusive. The dramatic shift in C/N metabolic partitioning resulting from defoliation and regrowth in forage grasses, such as *Lolium perenne* L., has the potential to reveal insights into the integration of C/N assimilation in plants, something not possible to observe in dicotyledonous species.

Fructans, polymers of fructose that are based on sucrose, are the major storage carbohydrate in temperate forage grasses, such as *L. perenne* (Turner *et al.*, 2006). We have previously confirmed that the fructan pool in *L. perenne* is degraded when NO_3_^–^ is supplied to N-deficient plants (Roche *et al.*, 2016), consistent with earlier work by Morvan-Bertrand *et al.* (1999) and Louahlia *et al.* (2008), and suggested that both root and shoot fructan pools support nitrate uptake and assimilation (Roche *et al.*, 2016). In response to defoliation, early shoot regrowth is also sustained by remobilization of the C stored as fructans in elongating leaf bases and mature leaf sheathes (Morvan-Bertrand *et al.*, 1999, 2001). Logically, the importance of root-derived C in supporting regrowth must also be considered in terms of the greater root biomass fraction that exists following defoliation. Thus, C and N remobilization under the new metabolic condition induced by defoliation must necessarily be co-ordinated at a whole-plant level.

The cytokinins, in addition to multiple roles in plant growth and development, are well known to play a role in regulating NO_3_^–^ uptake systems in response to fluctuations in N availability (Krouk *et al.*, 2011). The isoprenoid cytokinins are biosynthesized via adenosine phosphate-isopentenyltransferase (IPT). The first formed cytokinins are the nucleotides. The riboside forms are regarded as the main translocated forms, and the free bases the biologically active forms detected by receptors (Sakakibara, 2006; Lomin *et al.*, 2015). The signalling pathway in Arabidopsis involves a multi-step signalling network linking between the receptors and the response regulators (RRs) (Hwang *et al.*, 2012). The *cis*-forms of the cytokinins derive from the turnover of specific tRNA moities. Cytokinin homeostasis is determined by the rate of ctyokinin degradation which is catalysed by cytokinin oxidase/dehydrogenase (CKX) and by conjugation. For a general overview of the cytokinins, see Jameson (2017).

It has been shown that *trans*-zeatin (*t*Z)-type cytokinins (which carry a hydroxyl on the isoprenoid side chain) are the major forms in xylem sap, while isopentenyl adenine (iP-type) and *cis*-zeatin (*c*Z)-type cytokinins are the predominant forms in phloem sap (Takei *et al.*, 2004; Hirose *et al.*, 2008). Matsumoto-kitano *et al.* (2008) showed that iP-type cytokinins translocated from the shoot to the root in Arabidopsis were functional. It has been proposed that cytokinins could be involved in local and long-distance signalling to co-ordinate responses at the whole-plant level, and to communicate N status between multiple organs (Takei *et al.*, 2001; Sakakibara *et al.*, 2006; Matsumoto-Kitano *et al.*, 2008; Kudo *et al.*, 2010; Shtratnikova *et al.*, 2015). A split-root experiment in Arabidopsis has demonstrated that *NITRATE TRANSPORTERNRT2.1* and *NAR2.1* regulation, driven by shoot–root systemic N signalling, is mediated by cytokinin biosynthesis (Ruffel *et al.*, 2011). Further, genome-wide analyses for cytokinin-responsive genes in Arabidopsis have indicated that exogenous cytokinin can down-regulate root-localized transporters including *AtNRT1.1*, *AtNRT2.1*, *AtNRT2.2*, and *AtNRT1.5* (Brenner *et al.*, 2005; Li *et al.*, 2007). These gene families have been functionally identified as major components of the NO_3_^–^ uptake and xylem loading system (Liu *et al.*, 1999; Lin *et al.*, 2008). In contrast, cytokinin also up-regulates shoot-localized *AtNRT* genes under both high and low N conditions (Kiba *et al.*, 2011).

Additionally, global gene profiling coupled with physiological analysis in Arabidopsis has indicated that 74% of cytokinin-regulated genes may be significantly affected by glucose, either agonistically or antagonistically (Kushwah and Laxmi, 2014). Additionally, up-regulation of *AtIPT3,* and some Type A *ARABIDOPSIS RESPONSE REGULATOR* (*ARR*) genes and *CYTOKININ RESPONSE FACTOR* (*CRF*) genes by glucose suggests a potential role for glucose in modulating cytokinin signalling (Kushwah and Laxmi, 2014). It is thus plausible that cytokinins may play an active role in balancing the C–N status of the plant (Wang and Ruan, 2016).

Cytokinin deficiency, resulting from overexpression of *CKX*, in Arabidopsis causes an increase in the root/shoot ratio, with inhibited shoot growth and a larger root system (Werner *et al.*, 2003, 2008). This observation indicates that cytokinin deficiency may trigger a shift in C allocation from shoot to root, enhancing soil ‘foraging’ capacity for nitrate under limited N availability (Ruffel *et al.*, 2011). Once taken up, the increased NO_3_^–^ induces cytokinin biosynthesis in a nitrate-dependent manner (Krouk *et al.*, 2011). Subsequently, the cytokinins translocated to the shoot could regulate C allocation to keep shoot and root development in balance (Wang and Ruan, 2016).

With an ultimate goal of finding ways to better manage N input in grassland-based agricultural systems such as dairy farming, we investigated the nitrate uptake response to two independent but coinciding metabolic demands on fructan: defoliation and nitrate addition. Of particular interest was the degree of metabolic constraint, if any, that the defoliation–regrowth cycle and associated fructan depletion had on inorganic N uptake efficiency. Given the C cost associated with inorganic N uptake and assimilation (Roche *et al.*, 2016), we hypothesized that this metabolic demand is not adequately met in the days immediately following defoliation, due to regrowth competing with N uptake and assimilation for the stored C. To test this hypothesis, we carried out a stable isotope N uptake experiment, measured fructan content, and profiled cytokinin metabolites and relevant genes in *L. perenne* subjected to defoliation and glucose supplementation in a hydroponic system.

## Materials and methods

### Plant growth

Seeds of *L.perenne* L. cv. Grasslands Nui were germinated in Eppendorf tubes filled with perlite (tube tips removed) and the tubes placed in unfertilized soil for 11 weeks at 22°C. The basic N-free Hoagland medium (pH 6.0; Bioworld, USA) was supplemented with 0.05 mM or 5 mM KNO_3_ as a sole N source every week. Eight-week-old plants were defoliated at 4 cm above ground level. After 3 weeks regrowth, the plants were transferred to a hydroponic system which contained basic N-free Hoagland medium supplemented with either 0.05 mM or 5 mM KNO_3_, consistent with the previous growth conditions. The pH of treatment solutions was maintained at 6.0. Based on preliminary experiments, a 1 week adaptation phase in liquid culture medium was required for plants to retain pre-transfer competence (as assessed by leaf gas exchange and stomatal conductance measurements). After the 1 week adaptation, half the plants were again defoliated. The time zero of the experiments is defined by the second defoliation of plants.

### K^15^NO_3_ uptake measurement and isotope analysis

Root uptake of NO_3_^–^ was determined by ^15^N labelling. At 0 h and 48 h after defoliation, plants were gently blotted on tissue paper and then immediately rinsed with 0.1 mM CaSO_4_ for 1 min to remove any adsorbed compounds on the root surface, followed by 1 h exposure to basic N-free Hoagland medium supplemented with either 0.05 mM or 5 mM ^15^N-labelled KNO_3_ (atom % ^15^N: 10%). During the uptake experiments, the incubation solutions were aerated by an aquarium pump. At the end of the incubation period, roots were immediately rinsed with 0.1 mM CaSO_4_ for 1 min. Leaf sheaths and roots were separated, frozen in liquid nitrogen immediately, and stored at –80°C. The incubation timing was based on that used by Brackin *et al.* (2015). For this study, five plants were pooled as one biological replicate, and each treatment had five independent biological replicates. A 1 g aliquot of fresh samples was ground to a fine powder and freeze-dried for 3 d. Total N and ^15^N in the samples were determined with an isotope ratio mass spectrometer (Waikato University, New Zealand). A flow diagram of the experimental processes is shown in Supplementary Fig. S1 at *JXB* online.

### Glucose treatment

After 42 h of regrowth as described above, 100 plants grown in solutions with 5 mM KNO_3_ were then supplemented with either 0.1% or 1% (w/v) glucose (25 plants each) or with 0.1% or 1% (w/v) mannitol (25 plants each) as an osmotic control. After 6 h, K^15^NO_3_ uptake measurements were carried out by exposing roots to 5 mM ^15^N-labelled KNO_3_ (atom % ^15^N: 10%) solutions as described above. A flow diagram of the experimental processes is shown in Supplementary Fig. S2.

### Target gene sequence determination

Sequences of candidate target gene family members in perennial ryegrass were determined through BLAST searching the NCBI database and an RNA-Seq transcriptome database using prfectBLAST 2.0 software. An Illumina HiSeq2000 genome analyser (Macrogen Ltd, Seoul, Korea) and a pool of combined RNA samples extracted from multiple developmental stages of leaves, flower spikes, and seeds of perennial ryegrass cv. Nui were used to generate the transcriptome database containing 169 862 assembled sequence contigs of 595 bp in average length. All available orthologue sequences of the target gene families in the GenBank database for perennial ryegrass and closely related species were used as local BLAST search query sequences. The putative sequences of interest were verified via BLAST searching the GenBank database and via multiple sequence alignment with representative orthologue sequences in closely related species.

Neighbor–Joining (NJ) phylogenetic trees of the newly identified sequences of *LpNRT1*, *LpNRT2*, *LpNR*, *LpNiR*, *LpCKX*, *LpRR*, and *LpFEH*, and their orthologues were created using Clustal X software with 1000 bootstrap replicates. The phylogenetic trees were visualized with TreeView software (Supplementary Fig. S3). Each tree was rooted with an outgroup orthologue sequence from an unrelated species. The *LpRR* tree was shown in our previous study (Roche *et al.*, 2016). The GenBank accession numbers for the nucleotide sequences determined in this work are listed in Supplementary Table S1.

### RNA isolation and quantitative RT-PCR

Total RNA was extracted from up to 100 mg of frozen samples using an RNase Plant Mini Kit (Qiagen, Germany), following the manufacturer’s protocols. Three independent tissue samples (each comprising tissues from five plants) for each treatment were used as biological replicates. Up to 1 µg of extracted RNA was converted to cDNA using a QuantiTect Reverse Transcription Kit according to the manufacturer’s instructions (Qiagen).

The relative expression levels were determined using reverse transcription quantitative PCR (RT-qPCR). Specific PCR primers were designed for each family member of the target genes, using Primer Premier 6.20. Primer sequences for reference genes and target genes can be found in Supplementary Table S1. A volume of 15 µl was used for all qPCRs containing 1 µl of 10-fold diluted cDNA, the relevant primers, and home-made SYBR Green master mix (Song *et al.*, 2012). PCR products were Sanger-sequenced to confirm homology to genes which were already identified in NCBI gene databases. For RT-qPCR, three technical replicates for each of three biological replicates were carried out for each sample set. The relative expression (fold change) of each target gene was corrected using the geometric means of the two reference genes, *LpELONGATION FACTOR* (*eEF-1α*) and *LpGAPDH*, and calculated using the 2^–∆∆Ct^ method as described in previous studies (Schmittgen and Livak, 2008; Song *et al.*, 2015).

### Fructan analysis

Perennial ryegrass samples were harvested and immediately flash-frozen in liquid nitrogen. Ground and freeze-dried plant materials (25 mg) were weighed and placed into 2 ml Eppendorf tubes for fructan extraction. After addition of 750 µl of boiling milliQ water, samples were vortexed for 5 s, placed on a heating block at 90°C for 15 min, and then cooled down to room temperature and centrifuged at 20 000 *g* for 10 min. The supernatants were transferred to filter tubes, centrifuged at 20 000 *g* for 2 min, and transferred to LC vials. Extracts were analysed using an Agilent 1290 Infinity LC System (Agilent Technologies, Waldbronn, Germany) coupled to an Agilent 6550 Accurate-Mass QTOF LC-MS system with a dual Agilent Jet Stream source operating in negative mode. The QTOF was tuned for a mass range of 70–1700 *m/z*. A 2 µl aliquot of the sample was injected onto an Acquity UPLC HSS T3 C18 column (2.1 × 50 mm, 1.8 µm) combined with a 2.1 mm×5 mm, 1.8 µm VanGuard pre-column (Waters Corporation, Milford, MA, USA) held at 40°C. Compounds were eluted using the linear gradient reported in Roche *et al*. (2017). Data were collected in centroid mode with an acquisition rate of 4 scans s^−1^ and 1975 transients per spectrum.

### Endogenous cytokinin quantification

Extraction and qualification of cytokinins from ~3 mg DW samples were performed as described previously (Dobrev and Kamınek, 2002; Antoniadi *et al.*, 2015) using the LC-MS/MS system consisting of an ACQUITY UPLC^®^ System (Waters) and a Xevo^®^ TQ-S (Waters) triple quadrupole mass spectrometer (Svačinová *et al.*, 2012).

## Results

### Regrowth 48 h after defoliation

**Fig. 1.**
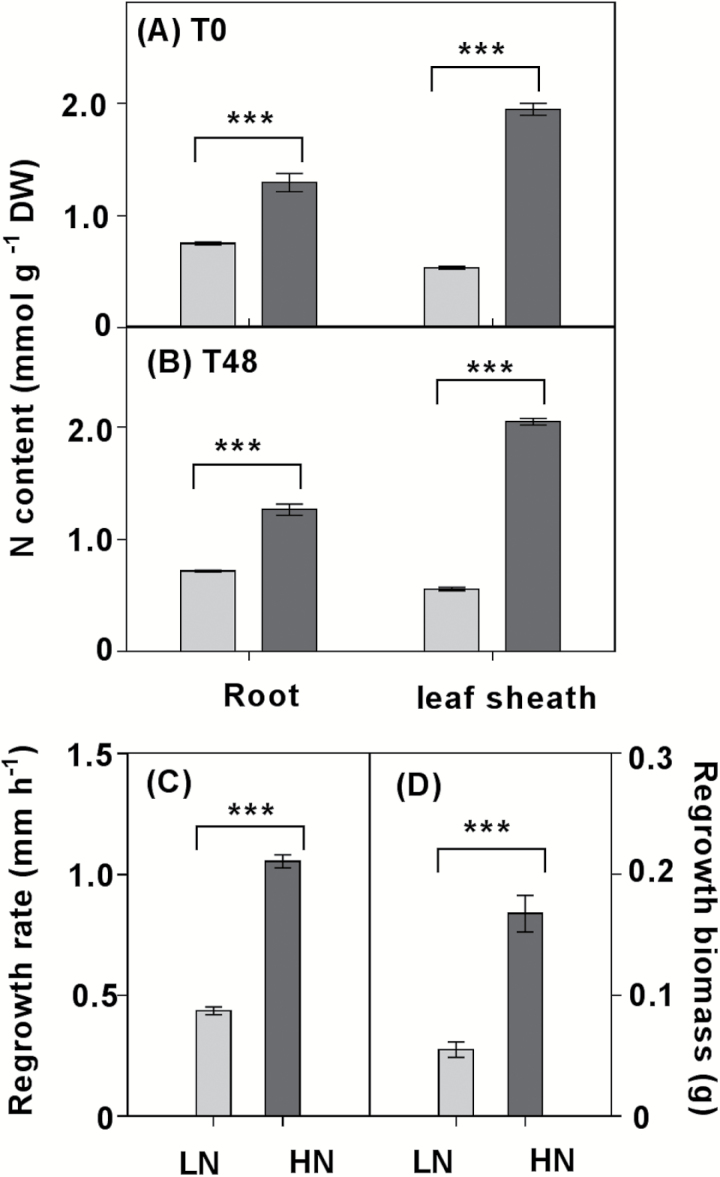
Growth parameters 48 h after defoliation of plants grown under low (0.05 mM) or high (5 mM) nitrate supply (LN versus HN). (A, B) Nitrogen (N) content before and 48 h after defoliation; (C) regrowth rate; (D) biomass 48 h after defoliation. Values in (A), (B), and (D) are means ±SE (*n*=5 pools of five plants each). Values in (C) are means ±SE (*n*=36 plants). Means were tested for significance using a two-tailed *t*-test. Asterisks denote significantly different means between LN plants (grey bars) and HN plants (black bars) at ***P<0.001.

N content was measured in roots and leaf sheaths of 12-week-old *L. perenne* plants 8 d after they were transferred from soil to a hydroponic solution and then defoliated to 40 mm above the crown. For the purpose of this study, the leaf sheath is defined as tissue from crown to 40 mm above, including elongating leaf bases and mature leaf sheaths, and this same part was harvested for the analysis in the intact plants. As expected, leaf sheath and root N content, regrowth rate, and biomass were significantly greater in plants grown under high NO_3_^–^ (HN, 5 mM) conditions than in plants grown under low NO_3_^–^ (LN, 0.05 mM) (Fig. 1).

### Water-soluble carbohydrates and fructan exohydrolase gene transcript levels

**Fig. 2.**
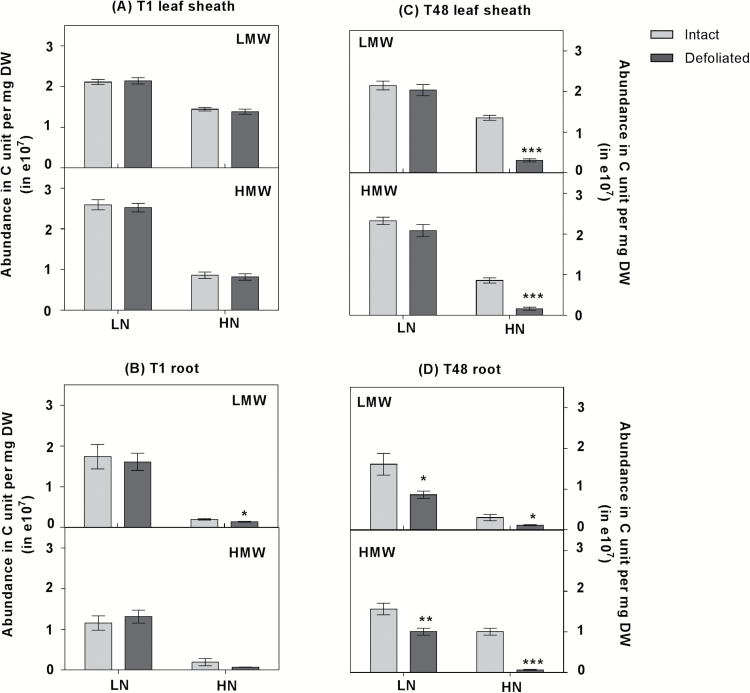
Abundance in carbon (C) units of water-soluble carbohydrates (WSCs). WSCs were measured in plants grown under high (HN) and low nitrate (LN) supply, 1 h and 48 h after defoliation. Relative abundance in C units was calculated by multiplying peak intensity by degree of polymerization (DP). WSCs with a DP from three to eight are referred to here as low molecular weight (LMW) WSCs, and DP9 to DP20 are referred to as high molecular weight (HMW) WSCs. The total concentrations of LMW and HMW WSCs are shown in (A) leaf sheaths and (B) roots after 1 h; (C) leaf sheaths and (D) roots after 48 h. Values are means ±SE (*n*=5 pools of five plants each). Means were tested for significance using a two-tailed *t*-test. Asterisks denote significantly different means between intact plants (grey) and defoliated plants (black) (*P<0.05, **P<0.01, ***P<0.001).

Water-soluble carbohydrates (WSCs) were profiled in order to assess the dynamics of C metabolism during the first 48 h of regrowth. WSCs with a degree of polymerization (DP) of 3–8 are referred to here as low molecular weight (LMW) WSCs, and those with DP9 to DP20 are referred to as high molecular weight (HMW) WSCs. With the exception of disaccharides, which showed similar relative abundance in LN and HN plants (Supplementary Fig. S4), LMW and HMW WSCs were found in greater abundance in LN roots and leaf sheaths compared with HN plants (Fig. 2). The detailed changes in the abundance of oligosaccharides with increasing DP are shown in Supplementary Fig. S4. During the first hour after defoliation, the remobilization of fructans occurred only in roots of HN plants, while no differences were observed in either roots or leaf sheaths of LN plants relative to intact plants (Fig. 2A, B; Supplementary Fig. S4). In comparison with the intact plants, the total concentration of LMW and HMW WSCs was significantly reduced 48 h after defoliation in both roots and leaf sheaths of HN plants, whereas in LN plants depletion of the fructan pool was only observed in roots, implying that the nature of defoliation-induced remobilization of the fructan pool after 48 h was tissue specific (Fig. 2C, D). In roots, the extent of exhaustion of the fructan pool of HN plants was more severe compared with that in LN plants (Fig. 2B, D; Supplementary Fig. S4).

*Lp1-FEH* and *Lp6-FEH* have been functionally characterized as fructan exohydrolases in *L. perenne* (Lothier *et al.*, 2007, 2014). Within 48 h of defoliation, *Lp6-FEH* expression was induced in leaf sheath and root of HN plants, and also in LN roots (Supplementary Fig. S5). Similarly, *Lp1-FEH* transcription in response to defoliation increased significantly in LN roots and leaf sheaths after 48 h, where the fructan content was highest (Fig. 2D; Supplementary Fig. S5).

### Nitrate uptake rate and N allocation

**Fig. 3.**
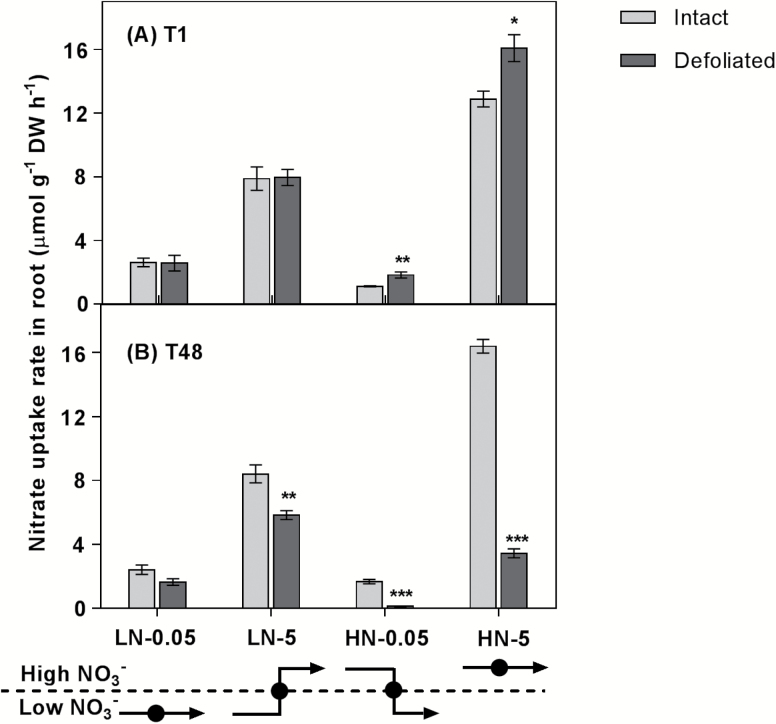
The impact of defoliation on NO_3_^–^ uptake in *L. perenne*. In order to investigate HATS and LATS uptake capacity, plants were grown under low NO_3_^–^ (LN: 0.05 mM) or high NO_3_^–^ (HN: 5 mM), and then defoliated (or left intact). Plants were then exposed for 1 h to either 0.05 mM or 5 mM ^15^N-labelled NO_3_^–^ either immediately after defoliation or 48 h after defoliation. During the period of ^15^N labelling, the NO_3_^–^ uptake rate was measured. Values are means ±SE (*n*=5 pools of five plants each). Means were tested for significance using a two-tailed *t*-test. Asterisks denote significantly different means between intact plants (grey bars) and defoliated plants (black bars) (*P<0.05, **P<0.01, ***P<0.001).

To better understand changes in NO_3_^–^ uptake in response to defoliation, plants were exposed to 0.05 mM and 5 mM NO_3_^–^ to stimulate either the nitrate high-affinity transport system (HATS) or the low-affinity transport system (LATS), respectively. The concentrations chosen were well below or above the point at which HATS or LATS would be saturated (Siddiqi *et al.*, 1990; Garnett *et al.*, 2013). During the first 1 h after defoliation, we observed no difference in NO_3_^–^ uptake rate via HATS or LATS in LN plants compared with intact plants, whereas NO_3_^–^ uptake via both HATS and LATS in defoliated HN plants was significantly greater relative to intact plants (Fig. 3A). In intact plants, HATS uptake in LN plants was greater than that in HN plants, while a much greater LATS uptake was induced in HN plants relative to LN plants (Fig. 3A). At 48 h after defoliation, the uptake rate of both HATS and LATS in HN plants dropped by 93% and 79% relative to intact plants, respectively (Fig. 3B). The HN plants, which had a relatively severe depletion of the fructan pool, showed markedly greater reduction in both HATS and LATS NO_3_^–^ uptake than that of LN plants 48 h after defoliation, implying a dependence on C for the uptake of nitrate.

To investigate the impact of defoliation on N distribution, ^15^N allocation to leaf sheaths following the incubations in ^15^N-labelled NO_3_^–^ was measured at 1 h and 48 h after defoliation. Relative allocation was determined as the proportion of labelled ^15^N in leaf sheaths to that in the total plant (roots and leaf sheaths). During the first hour after defoliation, relatively less ^15^N was allocated to leaf sheaths in defoliated LN and HN plants than in the controls (Supplementary Fig. S8). In contrast, despite the decline in NO_3_^–^ uptake rate 48 h after defoliation (Fig. 3B), the ^15^N allocation to leaf sheaths was relatively greater in both HN and LN defoliated plants than in intact plants (Supplementary Fig. S8).

### *NRT*, *NR*, and *NiR* transcript levels

**Fig. 4.**
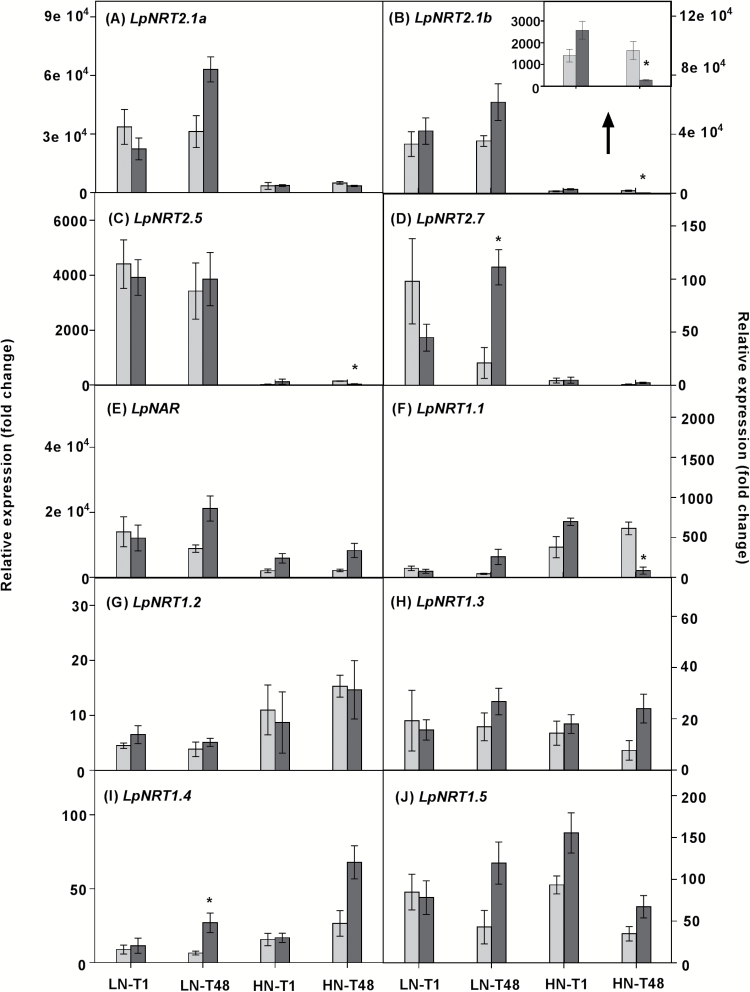
Expression of putative low- and high-affinity (*LpNRT1*, *LpNRT2*, and *LpNAR*) NO_3_^–^ transporter genes in roots of *L.perenne*. Plants were grown at either 0.05 mM (LN) or 5 mM (HN) NO_3_^–^, and then defoliated (or left intact). The expression level of *LpNRT2.1b* in HN roots is shown in the inserted bar graph in (B). Each data point is normalized against the reference genes *eEF-1α* and *GAPDH*. Values are means ±SE (*n*=3 pools of five plants each). Means were tested for significance using a two-tailed *t*-test. An asterisk denotes significantly different means between intact plants (grey bars) and defoliated plants (black bars) at *P*<0.05.

In our hydroponic system, we expected that HATS and LATS transcripts would be relatively abundant in LN and HN plants, respectively. Indeed, as expected, *NRT2* and *NAR* transcript levels in LN roots were generally greater than those in HN roots (Fig. 4A–E). This was particularly evident for the putative HAT candidate genes *LpNRT2.1a* and *LpNRT2.1b*, where transcript expression in roots of LN plants was 20- to 50-fold greater relative to HN plants (Fig. 4A, B). No significant difference in transcript abundance of *LpNRT2.1a* and *LpNRT2.1b* was observed between defoliated and intact LN plants during the first hour or 48 h after defoliation. Although transcript levels were relatively low in HN roots, a reduction in *LpNRT2.1b* transcript abundance was apparent 48 h after defoliation (Fig. 4B). Notably, within 1 h of defoliation, *LpNRT2.1b* expression was slightly but not significantly induced in HN roots (*P*=0.07; Fig. 4B). However, there was no significant difference in *LpNRT2.1a* transcription in HN roots following 48 h of defoliation (Fig. 4A). Despite their relatively lower presence, *LpNRT2.5* and *LpNRT2.7* showed greater expression in LN roots than in HN roots, suggesting that these two putative transporters may play a role in the HATS (Fig. 4C, D). Similarly to *LpNRT2.1a* and *LpNRT2.1b*, *LpNAR* displayed greater expression in LN roots relative to HN roots, confirming an important role for *LpNAR* in the function of NRT2.1 in NO_3_^–^ uptake (Fig. 4E).

Although the transcript levels of *LpNRT1.1* were relatively quite low, the expression pattern contrasts with those of the two putative HATS genes (*LpNRT2.1a* and *LpNRT2.1b*), with *LpNRT1.1* being generally higher in HN conditions than in LN conditions, suggesting that the putative *NRT1.1* may play an important role in the LATS (Fig. 4F). However, in HN roots, the transcript level of *LpNRT1.1* was reduced 48 h after defoliation, showing a similar pattern to that of *LpNRT2.1b* (Fig. 4F). Unlike *LpNRT1.1*, other *LpNRT1* genes, *LpNRT1.2*, *1.3*, *1.4*, and *1.5*, showed extremely low, but constitutive expression patterns regardless of treatment, indicating that these putative *NRT1* genes might be less sensitive to changing N conditions (Fig. 4G–J).

**Fig. 5.**
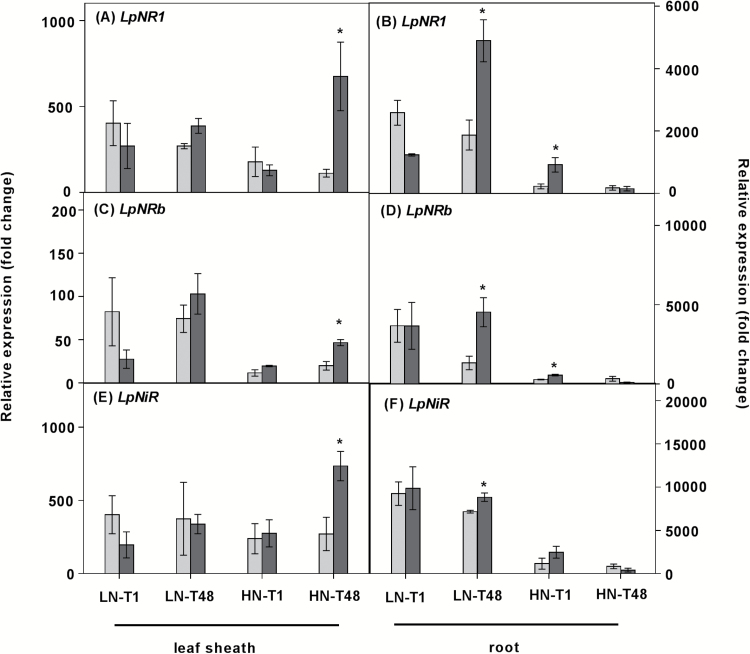
Expression of putative nitrate reductase (*LpNR*) and nitrite reductase (*LpNiR*) genes in plants. Plants were grown at either 0.05 mM (LN) or 5 mM (HN) NO_3_^–^, and then defoliated (or left intact). Each data point is normalized against the reference genes *eEF-1α* and *GAPDH*. Values are means ±SE (*n*=3 pools of five plants each). Means were tested for significance using a two-tailed *t*-test. An asterisk denotes significantly different means between intact plants (grey bars) and defoliated plants (black bars) at *P*<0.05.

In order to determine whether N assimilation is tightly co-ordinated, we assessed the influence of the C/N balance on the expression of nitrate reductase (*NR*) and nitrite reductase (*NiR*) genes. Similar to nitrate transporter gene expression, transcript levels of genes involved in nitrate assimilation (*LpNR1*, *LpNRb*, and *LpNiR*) were more abundant in plants grown under LN conditions relative to those under HN conditions (Fig. 5). With exposure to HN conditions, expression of *LpNR1* and *LpNRb* in sheaths was significantly induced 48 h after defoliation, whereas *LpNR* expression in roots showed no significant difference 48 h after defoliation (Fig. 5A–D). Notably, in HN roots, expression of *LpNR1* and *LpNRb* was induced during the first hour after defoliation (Fig. 5B, D), which is consistent with the stimulation of NO_3_^–^ uptake in HN plants following 1 h defoliation (Fig. 3A). The transcripts of *LpNiR* showed a similar pattern to that of *LpNR1* and *LpNRb*. In LN conditions, expression of *LpNR1*, *LpNRb*, and *LpNiR* was induced in roots 48 h after defoliation, whereas there was no change in leaf sheaths (Fig. 5B, D, F).

### Glucose supplement

**Fig. 6.**
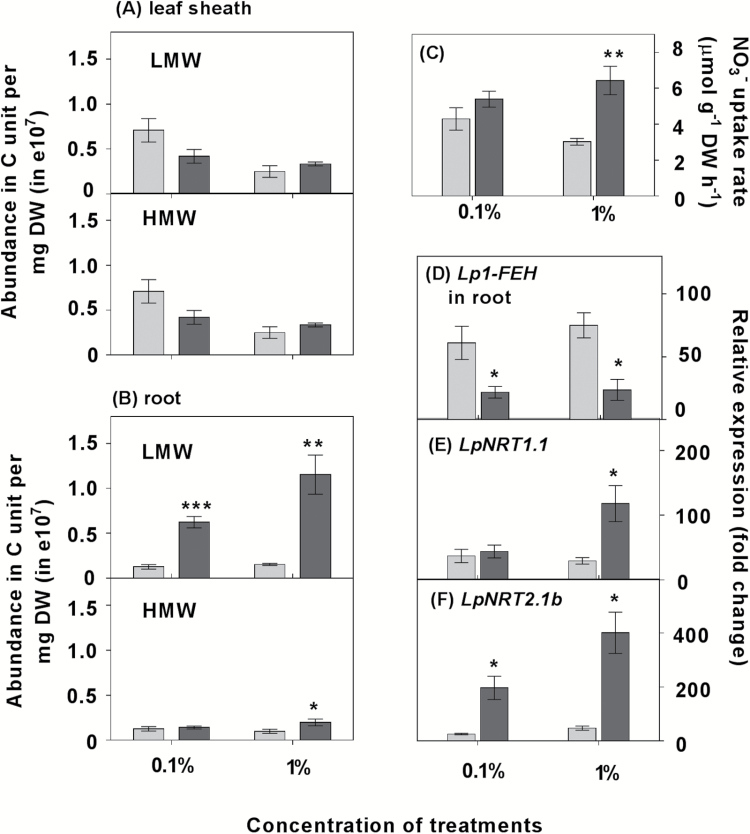
WSC content, NO_3_^–^ uptake, and *Lp1-FEH*, *LpNRT1.1*, and *LpNRT2.1b* expression in roots with 6 h of supplemental glucose. Plants were grown in HN conditions and supplied with 0.1% or 1% glucose 42 h after defoliation. After 6 h, the impact of supplemental 0.1% or 1% glucose treatment on: (A, B) the relative abundance (in carbon units) of water-soluble carbohydrate (WSC); (C) NO_3_^–^ uptake rate; and (D–F) *Lp1-FEH*, *LpNRT1.1*, and *LpNRT2.1* expression in roots. Values are means ±SE (*n*=5 pools of five plants each). Means were tested for significance using a two-tailed *t*-test. Asterisks denote significantly different means between mannitol (grey) and glucose (black) treatment (*P<0.05, **P<0.01, ***P<0.001).

To distinguish further the relationship between carbon change dynamics following defoliation and N uptake responses, a subset of the HN plants were subjected to exogenous glucose treatment 42 h after defoliation. After 6 h of treatment with glucose, fructan abundance in roots increased compared with the mannitol control (Fig. 6B), whereas there was no significant difference in fructan abundance in the leaf sheaths (Fig. 6A). The detailed changes in the abundance of oligosaccharides with increasing DP are shown in Supplementary Fig. S6. Glucose supplementation led to repression of *Lp1-FEH* and *Lp6-FEH* expression in the roots but not in the leaf sheaths (Fig. 6D; Supplementary Fig. S7).

After 6 h of glucose treatment, a subset of HN defoliated plants was moved to 5 mM ^15^N isotopically labelled NO_3_^–^ for NO_3_^–^ uptake measurement. NO_3_^–^ uptake increased significantly in the plants exposed to 1% glucose compared with those in the mannitol treatment (Fig. 6C). The 1% glucose supplement induced a significant decline in the ^15^N allocation to leaf sheaths (Supplementary Fig. S8).

Expression of both *LpNRT2.1b* and *LpNRT1.1* was significantly enhanced by exposure to glucose (Fig. 6E, F). There was no significant change in the transcript abundance of *LpNRT2.1a* following glucose treatment (Supplementary Fig. S9).

### Changes in endogenous cytokinin content following defoliation

**Fig. 7.**
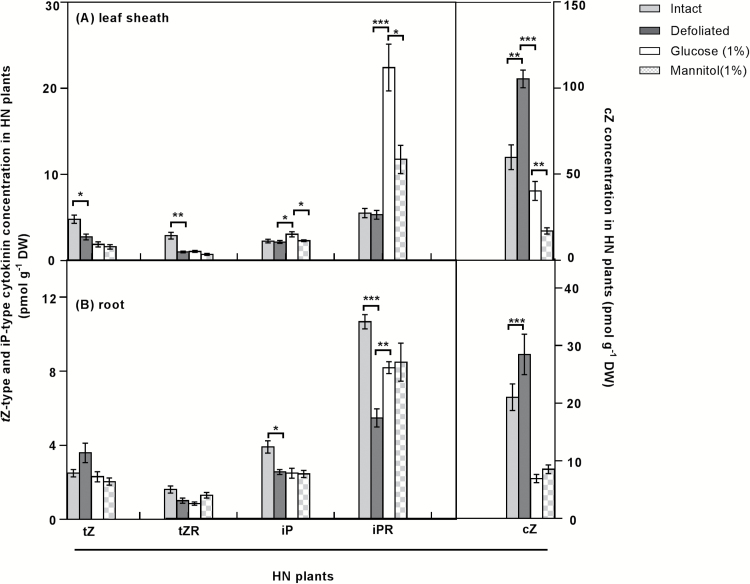
Cytokinin concentrations in high N (HN) roots and leaf sheaths 48 h after defoliation. Values are means ±SE (*n*=5 pools of five plants each). Asterisks denote significantly different means between intact plants (grey bars) and defoliated plants (black bars), or between defoliated plants (black bars) and defoliated plants supplemented with 1% glucose (white bars) or 1% mannitol (hatched bars) (*P<0.05, **P<0.01, ***P<0.001). Means were tested for significance using a two-tailed *t*-test.

To better understand the role of cytokinin in communicating the root–shoot C/N balance at the whole-plant level, cytokinins were quantified in *L. perenne* plants. Overall, the total cytokinin complement was greater in the leaf sheaths and roots of HN plants compared with LN plants, irrespective of defoliation. Under HN, both *t*Z and *t*Z riboside (*t*ZR) had decreased in the leaf sheaths 48 h after defoliation, whereas both iP and iP riboside (iPR) decreased in the roots 48 h after defoliation, relative to intact controls (Fig. 7). In the LN plants, with the exception of an accumulation of iPR in leaf sheaths 48 h after defoliation, there was little change in other cytokinins (Supplementary Fig. S10). In addition, iP and iPR in leaf sheaths was greater in HN defoliated plants following the 6 h 1% glucose treatment relative to HN defoliated plants without glucose treatment. Although there was no significant difference in the iPR content between the glucose and mannitol treatments, the iPR content was greater in HN roots with 1% glucose treatment relative to HN defoliated roots without glucose or mannitol addition (Fig. 7). Substantial amounts of *c*Z-type cytokinins were detected in HN plants. Among them, *c*Z accumulated in leaf sheaths in the 48 h following defoliation of HN plants, compared with the leaf sheaths of intact plants (Fig. 7). Further, *c*Z content decreased in leaf sheaths and roots of plants treated with glucose relative to HN defoliated plants without glucose or mannitol treatment. However, this decrease was also observed in the 1% mannitol treatment (Fig. 7).

### Expression of cytokinin oxidase/dehydrogenase and cytokinin response regulator gene family members.

**Fig. 8.**
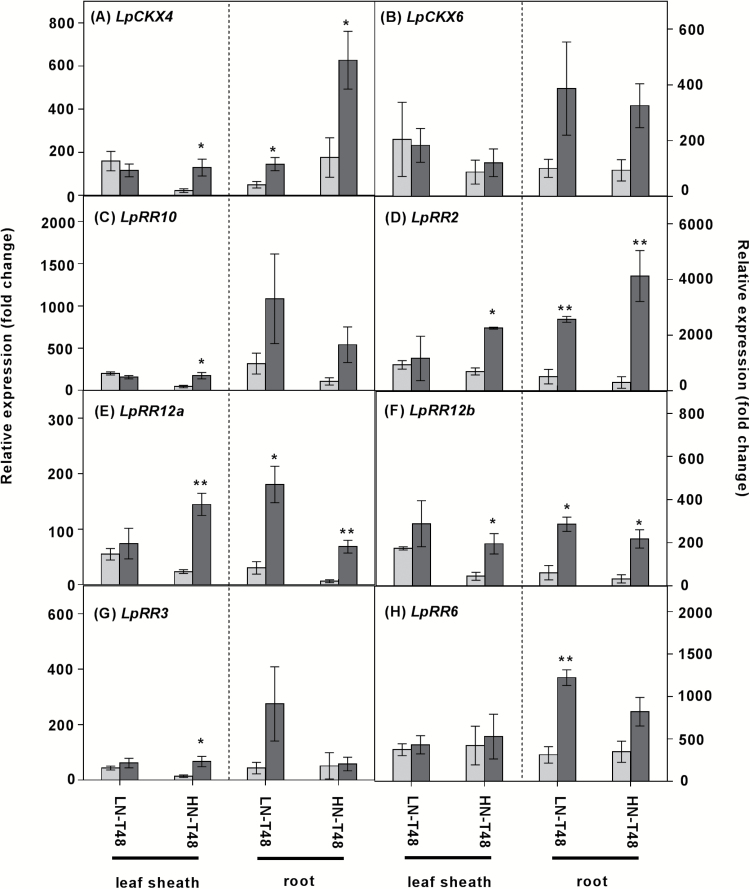
The impact of 48 h defoliation on the putative *LpCKX* and *LpRR* gene expression. Plants were grown at either 0.05 mM (LN) or 5 mM (HN) NO_3_^-^, and then defoliated (or left intact). Each data point is normalized against the reference genes *eEF-1α* and *GAPDH*. Values are means ±SE (*n*=3 pools of five plants each). Means were tested for significance using a two-tailed *t*-test. Asterisks denote significantly different means between intact plants (grey bars) and defoliated plants (black bars) (*P<0.05, **P<0.01).

In roots, significant *LpCKX4* induction was observed in LN and HN plants 48 h after defoliation (Fig. 8A), and in HN leaf sheaths 48 h after defoliation, relative to intact plants (Fig. 8A). No significant difference was observed during the first hour after defoliation, when compared with intact plants (Supplementary Fig. S11).

**Fig. 9.**
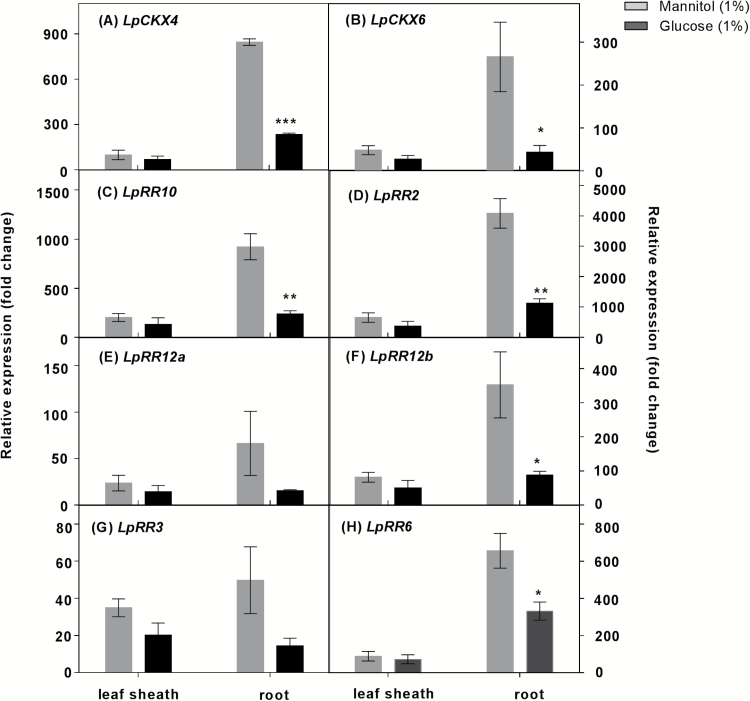
Effects of addition of 1% glucose on putative *LpCKX* and *LpRR* gene expression in plants grown in 5 mM NO_3_^–^, 48 h after defoliation. Each data point is normalized against the reference genes *eEF-1α* and *GAPDH*. Values are means ±SE (*n*=3 pools of five plants each). Means were tested for significance using a two-tailed *t*-test. Asterisks denote significantly different means between mannitol (grey bars) and glucose (black bars) treatment (*P<0.05, **P<0.01, ***P<0.001).

 In comparison with intact plants, increases in expression of the four Type-B *RR* genes (*LpRR2*, *LpRR10*, *LpRR12a*, and *LpRR12b*) and two Type-A *RR* genes (*LpRR3* and *LpRR6*) occurred in leaf sheaths of HN plants 48 h after defoliation, whereas no significant differences were displayed in leaf sheaths of LN plants (Fig. 8C–H). In roots, transcript levels of *LpRR* genes were greater in both LN and HN conditions 48 h after defoliation (Fig. 8C–H). Expression of *LpCKX4*, *LpCKX6*, and *LpRR* genes was strongly reduced in roots following the 1% glucose treatment of HN defoliated plants (Fig. 9A–F, H). Under 0.1% glucose treatment, there was no significant difference in expression of these gene families (Supplementary Fig. S12).

## Discussion

An understanding of the mechanisms linking C/N balance to N uptake and assimilation is vital for optimizing plant growth in pasture-based production systems where defoliation by grazing is followed by N fertilizer addition. As shown previously, the fructan carbohydrate pool in elongating leaf bases and sheaths may be severely depleted in order to sustain regrowth following the removal of photosynthetically active tissues in grasses such as *L. perenne* (Morvan-Bertrand *et al.*, 1999, 2001). Here, by experimentally defoliating plants to interfere with the C/N balance and by mitigating this with exogenous glucose supplementation, we have uncovered the dynamic relationships between the carbohydrate status of the plant, the nitrate-responsive uptake system, transporter gene expression, and assimilation. We confirm and extend previous studies (Louahlia *et al.*, 1999) by showing that NO_3_^–^ HATS and LATS uptake changes in response to the rapid and large shift in C status induced by defoliation. We also explored the involvement of cytokinins in the regulation of the C/N balance in response to defoliation. The results are discussed in relation to the internal C/N balance under defoliation.

### Nitrate uptake and assimilation rely on labile carbohydrate availability

Consistent with previous studies (Louahlia *et al.*, 2008; Roche *et al.*, 2016), long-term N limitation led to significant accumulation of fructans in both roots and shoots relative to N-sufficient plants, which is important in the context of this study (Fig. 2). Two days after defoliation, severe fructan depletion occurred in both roots and leaf sheaths of HN plants. However, in LN plants, this depletion was only significant in roots, and was incomplete. We suggest that sufficient N in the HN plants was a stimulating factor in remobilization of the available fructan pool in leaf sheaths, which provided the required energy and C skeletons for regrowth. However, efficient regrowth following defoliation requires balanced pools of stored C for energy, cell biosynthesis, and C skeletons for nitrate reduction, together with stored N. LN plants clearly had insufficient N to utilize the stored C efficiently. These observations indicate that carbohydrate remobilization to support regrowth occurs in an N-dependent manner.

The regulation of root nitrate uptake by C metabolites from photosynthesis has previously been demonstrated through studying the diurnal stimulation of NO_3_^–^ uptake. Sugars transported from shoots to roots played an important role in this diurnal stimulatory effect (Rideout and Raper, 1994; Delhon *et al.*, 1996; Lejay *et al.*, 1999). Using defoliation as a treatment in the present study allowed us to reveal the response of root NO_3_^–^ uptake to changes in endogenous C. During the first hour following defoliation, we observed the immediate depletion of the fructan pool in roots of HN plants (Fig. 2B). This immediate remobilization of the fructan pool in HN roots was associated with a rapid and significant stimulation of root NO_3_^–^ uptake rate (Figs 2B, 3A). Given that there is a substantial amount of sucrose in phloem immediately after defoliation (Morvan-Bertrand *et al.*, 2001), this stimulation of NO_3_^–^ uptake by the root may be attributed to the initial increase in sucrose followed by fructan remobilization in roots, suggesting an instantaneous response of root NO_3_^–^ uptake to C availability. In contrast, 48 h after defoliation, low- and high-affinity NO_3_^–^ uptake decreased following depletion of the fructan pool in the roots, irrespective of the N status of the plants (Figs 2, 3). Interestingly, a relatively greater fructan depletion in HN plants than in LN plants was associated with a much stronger repression of nitrate uptake rate in HN plants than in LN plants, indicating that NO_3_^–^ uptake is a C-dependent process. These observations strongly suggest that root NO_3_^–^ uptake is modulated by C metabolites at the whole-plant level, which is consistent with the suggestion that photosynthate supply is closely balanced with mineral uptake and assimilation (Lejay *et al.*, 1999; Coruzzi and Zhou, 2001; Nunes-Nesi *et al.*, 2010).

### NO_3_^–^ assimilation is regulated by the C–N status of the plant

According to Scheurwater *et al.* (2002), the intact shoot of the eight grass species that they tested was the predominant site for nitrate reduction. This can be explained by the fact that nitrate assimilation is more efficient in the shoot when leaves are photosynthesizing in fully saturating light conditions. Under these conditions, nitrate assimilation in roots is inefficient because sucrose has to be synthesized, transported to roots, and respired in order to generate ATP and NAD(P)H for N assimilation, rather than being directly utilized in the shoot (Nunes-Nesi *et al.*, 2010). Our results also support tissue-specific N assimilation being regulated by local C availability. With relative high C availability in HN roots 1 h after defoliation, NO_3_^–^ uptake was induced instantaneously compared with intact plants (Fig. 3A). This induction of nitrate uptake in HN roots was concomitant with stimulation of *LpNR1* and *LpNRb* expression in HN roots (Fig. 5B, D). Given that light was non-saturating for expanding leaf tissue immediately following defoliation, because the leaf tissue was shaded by the sheath, we suggest that a relatively greater proportion of NO_3_^–^ was assimilated locally in HN roots during the first hour after defoliation. However, in the days (48 h) after defoliation, when root C was reduced in HN plants and the young leaves were emerging, relatively more N was allocated to shoots compared with intact plants, thereby stimulating shoot growth (Fig. 2D; Supplementary Fig. S8). This was concomitant with changes in the transcript levels of nitrate reductase genes. In HN plants, expression of *LpNR* and *LpNiR* in leaf sheaths was induced following the strong C remobilization in roots, whereas expression of *LpNR* and *LpNiR* was repressed in roots (Figs 2C, D, 5). These results suggested that a shift in nitrate assimilation from roots to shoots occurred when root carbon availability was reduced, supporting the suggestion that local C availability regulates the tissue-specific assimilation of N.

With low N status associated with a high C/N ratio, the transcripts of *LpNR* and *LpNiR* genes were more abundant in roots compared with leaf sheaths (Figs 2C, D, 5). We suggest that under low NO_3_^–^ availablility, roots with high C/N had sufficient carbohydrate to assimilate most of the acquired NO_3_^–^ into amino acids locally, and thus little N was allocated to shoots in LN plants, thereby potentiating root growth and root foraging. Interestingly, 48 h after defoliation, the significant C remobilization in LN roots was accompanied by a strong induction of *LpNR* and *LpNiR* expression in roots, whereas there was no change in the fructan pool and the expression of *LpNR* and *LpNiR* in leaf sheaths (Figs 2C, D, 5). These observations imply a mechanism that signals the availability of C metabolites for NO_3_^–^ uptake and assimilation at the whole-plant level. Within tissues, where stored C is available, it is apparent that NO_3_^–^ is assimilated locally.

### HATS and LATS regulation at the transcriptional level in response to a changing C/N ratio

Four *NRT* families have been characterized as being involved in nitrate uptake in Arabidopsis (Tsay *et al.*, 2007). Among them, *NRT1* and *NRT2* are regarded as the main components controlling the HATS and LATS that function at low (<250 µM) or high (>1 mM) nitrate levels, respectively (Siddiqi *et al.*, 1990; Tsay *et al.*, 1993; Huang *et al.*, 1999; Y.Y. Wang *et al.*, 2012). In our study, as expected, the expression of *LpNRT1.1* and *LpNRT2.1b* was significantly repressed in HN plants 48 h after defoliation, coinciding with reduced NO_3_^–^ uptake activity (Figs 3B, 4B, F). Interestingly, within 1 h of defoliation, the increased expression of *LpNRT2.1b* in HN roots (*P*=0.07) also coincided with the rapid induction of NO_3_^–^ uptake activity (Figs 3A, 4B). This is consistent with observations that regulation of root NO_3_^–^ uptake is correlated with changes in transcript levels of *NRT2.1* in response to N and C treatment (Zhuo *et al.*, 1999; Lejay *et al.*, 2003).

Conversely, but as expected, this was not the case in LN status plants which showed lower reductions in NO_3_^–^ uptake rate following defoliation relative to HN plants, and only small increases in both *LpNRT1.1* and *LpNRT2.1* expression (Figs 3B, 4B, F). Indeed, the regulation of high-affinity nitrate uptake is complicated in low nitrate conditions. This result is consistent with previous studies showing that large and rapid increases in HATS uptake in low NO_3_^–^ conditions are not accompanied by increased *AtNRT2.1* expression (De Jong *et al.*, 2014). One possible interpretation for the discrepancy between *NRT2.1* transcript and nitrate uptake involves the potential for post-transcriptional regulation of existing transporter levels and activity, for example by phosphorylation (Laugier *et al.*, 2012; X. Wang *et al.*, 2012).

### Exogenous glucose mitigates reduced nitrate uptake rate in defoliated plants

Exogenous glucose supplied for 6 h to defoliated HN plants largely restored LMW WSC levels in roots, which appears to have relieved the C limitation on NO_3_^–^ uptake and assimilation (Fig. 6B). The NO_3_^–^ uptake rate in defoliated plants supplemented with glucose attained a level greater than that in plants maintained in 5 mM NO_3_^–^ but without glucose supply. In contrast, application of 1% mannitol had no impact on the NO_3_^–^ uptake rate compared with plants kept in 5 mM NO_3_^–^, confirming the carbohydrate dependence of nitrate uptake as described above (Figs 3B, 6C). Interestingly, increasing C availability in roots was correlated with a decline in N allocation to leaf sheaths, which supports our observations of C and N interactions in nitrate uptake and metabolism (Fig. 6B; Supplementary Fig. S8).

The NO_3_^–^ uptake rescue by glucose was positively correlated with the transcript levels of *LpNRT1.1* and *LpNRT2.1b* (Fig. 6C, E, F), genes that are regarded as the major players in the LATS and HATS for nitrate, respectively (Y.Y. Wang *et al.*, 2012). Glucose and sucrose have been found to regulate the expression of genes involved in metabolism and nutrient uptake (Li *et al.*, 2006; Lejay *et al.*, 2008). However, up-regulation of *AtNRT2.1* by sugars is not attributed to well-established specific sucrose or glucose sensing, although, as shown by De Jong *et al.* (2014), glucose-regulated expression of *AtNRT2.1*, independent of nitrate-mediated mechanisms, can operate through HEXOKINASE1-mediated oxidative pentose phosphate pathway (OPPP) metabolism.

### Cytokinin is involved in the regulation of N acquisition and assimilation in response to changes in C/N

Cytokinins have been proposed as candidate signalling molecules relaying N status between the root and shoot (Kudo *et al.*, 2010; Ruffel *et al.*, 2014). We show here that the cytokinin content of intact HN status plants is greater than that in plants of LN status (Fig. 7; Supplementary Fig. 10). This is consistent with the observations that N supply leads to cytokinin accumulation in roots, xylem sap, and shoots in maize (Takei *et al.*, 2001), suggesting that cytokinin serves to signal N availability from the root to shoot (Sakakibara *et al.*, 2006). After defoliation under HN, a decrease was observed in the *t*Z-type cytokinin in the leaf sheaths and the iP-type cytokinin in the roots which was associated with increased *CKX* expression (Figs 7, 8A, B). Interestingly, 1% glucose addition to defoliated HN plants induced iP and iPR accumulation in leaf sheaths and roots, and was associated with a significant decline in expression of *LpCKX4* and *LpCKX6* in roots, implying that iP-type cytokinins might be an integrating signal of the relative availability of C and N metabolites in plants (Figs 7, 9A, B).

In contrast, *c*Z-type cytokinins increased in the HN leaf sheaths, as did expression of the *LpRR* genes, 48 h after defoliation (Figs 7, 8). *c*Z-type cytokinins are recognized by receptors and activate response regulators in rice and maize (Veach *et al.*, 2003; Yonekura-Sakakibara *et al.*, 2004; Lomin *et al.*, 2011; Roche *et al.*, 2016). In this context, it is possible that the *LpRR* genes could be responding to the *cis*-cytokinins with the downstream activation of N assimilation in the leaf sheaths. Consistently, while *c*Z-type cytokinin content declined, *LpRR* expression was reduced in roots following 6 h of 1% glucose addition (Figs 7, 9). However, this decline of *c*Z also occurred in plants with 1% mannitol treatment (Fig. 7). The decreased *c*Z content under 1% mannitol treatment was not consistent with salt or osmotic stress which is suggested to induce *c*Z (Havlová *et al.*, 2008; Vyroubalová *et al.*, 2009; Macková *et al.*, 2013). The *c*Z response to mannitol reflects the less well understood biology of the *cis*-cytokinin forms. As the *LpRR* expression decreased under glucose treatment but not under mannitol treatment, a cytokinin response to mannitol is not supported. Taken together with the increased NO_3_^–^ uptake rate, fructan levels, and *RR* gene profiles under glucose treatment, the glucose supplement may have caused a cytokinin response via its impact on C metabolism.

### Conclusions

**Fig. 10.**
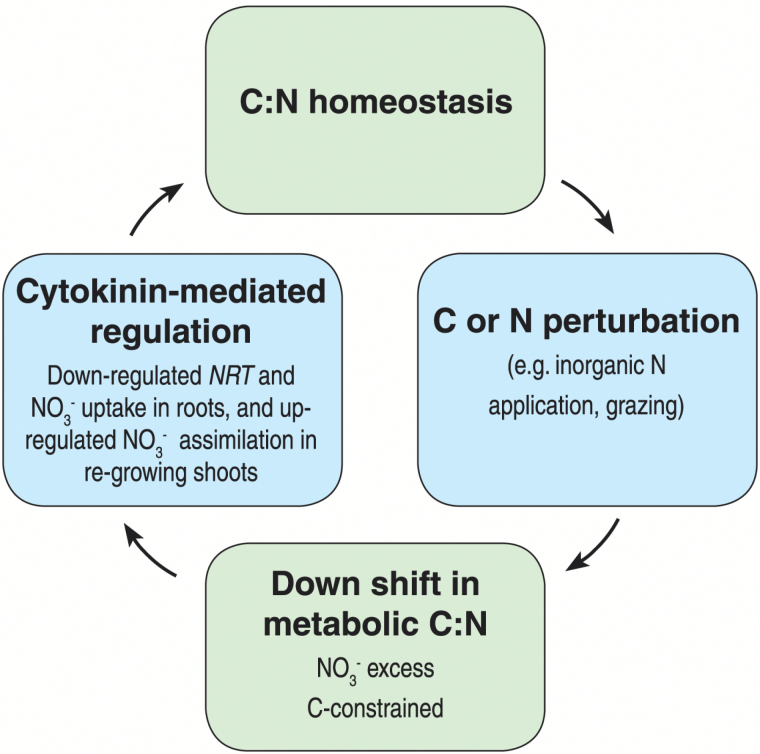
A proposed model depicting the role of cytokinin in C:N homeostasis. Cytokinin restores the C:N balance by down-regulating NO_3_^–^ uptake in a C-status-dependent manner: when grazing or inorganic N application leads to a decrease in the C:N balance, cytokinin serves as a signal to mediate the down-regulation of NO_3_^–^ uptake in roots and the up-regulation of NO_3_^–^ assimilation in the re-growing shoots, thereby re-balancing the C and N resources at the whole-plant level.

This study provides clear evidence that the rapid and large shifts in C storage triggered by defoliation have significant impact on nitrate uptake in an N-status-dependent manner. Our results for the perennial grass, *L. perenne*, are consistent with those in Arabidopsis, where cytokinins act as a systemic N signal regulating N uptake and assimilation (Kiba *et al.*, 2011; Ruffel *et al.*, 2011; Shtratnikova *et al.*, 2015). By perturbing C assimilation and metabolism with a defoliation–regrowth treatment, we propose that cytokinin-mediated NO_3_^–^ uptake and assimilation may also act to balance C and N resources at the whole-plant level. Building on our results and those of previous workers, we present a model (Fig. 10) whereby cytokinins serve as a signal to integrate C and N metabolism via tissue-specific function: cytokinin-mediated reduction in NO_3_^–^ uptake in roots and an increase in NO_3_^–^ assimilation in re-growing shoots under low C/high N status (low N demand); and a concomitant shift of C allocation from shoots to roots which serves to stimulate NO_3_^–^ assimilation in roots under high C/low N status (high N demand) (Fig. 10). As we develop our understanding of the interaction between defoliation and N uptake, this knowledge may be applied to pasture production for grazing in order to increase N uptake and utilization efficiency.

## Supplementary data

Supplementary data are available at *JXB* online.

Table S1. Sequences of qPCR primers used in this work.

Figs S1 and S2. Flow diagrams of the experimental processes

Fig. S3. Phylogenetic trees of *LpNRT1*, *LpNRT2*, *LpNR*, and *LpNiR* gene families.

Fig. S4. Relative abundance in carbon (C) units of water-soluble carbohydrates (WSCs) in HN and LN plants after defoliation

Fig. S5. Expression of putative *Lp6-FEH* and *Lp1-FEH* in plants grown under either 0.05 mM (LN) or 5 mM NO_3_^–^ (HN) supply, 1 h and 48 h after defoliation.

Fig. S6. The impact of glucose on relative abundance in carbon (C) units of water-soluble carbohydrates (WSCs).

Fig. S7. *Lp6-FEH* and *Lp1-FEH* expression in leaf sheaths with 6 h of supplemental glucose.

Fig. S8. The impact of defoliation or glucose addition on ^15^N translocation to shoots.

Fig. S9. Effects of glucose addition on putative *LpNRT2.1a* gene expression in roots grown in 5 mM NO_3_^-^, 48 h after defoliation.

Fig. S10. Cytokinin concentrations in LN roots and leaf sheaths 48 h after defoliation.

Fig. S11. The impact of 1 h defoliation on putative *LpCKX* and *LpRR* gene expression.

Fig. S12. Effects of 0.1% glucose addition on putative *LpCKX* and *LpRR* gene expression in plants grown in 5 mM NO_3_^–^, 48 h after defoliation.

## Supplementary Material

Supplementary_Table_S1_Figures_S1_S12
