# Supplementary material for: Depletion of carbohydrate reserves limits nitrate uptake during early regrowth in Lolium perenne L
Source: J Exp Bot. 2017 Mar 31;68(7):1569–83. doi: 10.1093/jxb/erx056 (PMC5444434; doi:10.1093/jxb/erx056)
Supplement: Supplementary_Table_S1_Figures_S1_S12 [file erx056_suppl_Supplementary_Table_S1_Figures_S1_S12.pdf]

Supplementary Data

Table S1. **Primer sequences**

F, forward; R, reverse

**Primers**	**Sequence**	**GenBank Accession No.**
Lp1-FEHF	AAGGTGCCAAACATGTCCTC	AY693396.5
Lp1-FEHR	TGCGACGTCATCTGAAGAAC	
Lp6-FEHF	AGATAGTGTTTCAGATGATGGCGTG	KY554803
Lp6-FEHR	CATGCTCCATGCCCTGAGTTG	
LpNRT1.1F	CGTACATCGGVCAGCTMGACTTCT	KY554804
LpNRT1.1R	YTGATGTCGTCGGCGAGCCAG	
LpNRT1.2F	TCAGCAGCATTGGTGGAGAGTAAC	KY554805
LpNRT1.2R	GCACAAGAGAMAGGATRAACGAAY	
LpNRT1.3F	CCTCCTCTTCACCTCCCTCAACGAG	KY554806
LpNRT1.3R	AACTGCGGCACCAGCCAGAAC	
LpNRT1.4F	GGACCATCTACGCGCAGATGAT	KY554807
LpNRT1.4R	ACGAGGCCGATGCCGATCTTCTC	
LpNRT1.5F	CCWYMGCCCTGCTGCTSTTC	KY554808
LpNRT1.5R	AACCTGAAGCCTTYGGTGTGCAGAAG	
LpNRT2.1aF	CACCTCTCGTGGATCTCCTTCTT	KY554809
LpNRT2.1aR	GCGGCGAGCATGACGAGGAAG	
LpNRT2.1bF	GCTGGTGGTAACGTGGGTGCAG	KY554810
LpNRT2.1bR	AGCGGGAGTTCTCKGCRAACTTTTG	
LpNRT2.5F	CTGCCGCTCATCCGGGACAC	KY554811
LpNRT2.5R	MGKYGATGATGGASGAGCAGTACAC	
LpNRT2.7F	CATCCCCTGCGCCCTGCTCATC	KY554812
LpNRT2.7R	GCGGCCACGTTYTCCATGATGAG	
LpNARF	CGAGCACAAGGCSAAGWCCW	KY554813
LpNARR	AGCGGGAGTTCTCTGCGAACT	
LpNR1F	CCGTCCATGGCGGTTCCAATC	KY554814
LpNR1R	CCGGAGCCGTACTCGTC	

LpNRbF	CCTCACCGCCATCGTCGA	KY554815
LpNRbR	CAGGCCGACGAAGGAG	
LpNiRF	AGAGCAGCCACGCCGAC	KY554816
LpNiRR	ACACRAAGATGTGCTTG	
LpCKX4F	CTGAACCTTCTWATCCCAAGAAGC	KY554817
LpCKX4R	CAGGAGGCGTCCTTCGGTTTCA	
LpCKX6F	AGTTTGCGGTTCATGATTACCAGCAC	KY554818
LpCKX6R	CGCCCACGGAGAGGTAGAGGTAG	
***LpRR2*F**	CCAGTTCGTCTAGCTTTCAGAGTTCC	KU136271
***LpRR2*R**	GCCTTCACATCTGTCCACTAAATCCG	
***LpRR3*F**	ATCGTCGGAGCTGAAGCAGATTC	KU136272
***LpRR3*R**	CTGACAGGCTTGAGCAGGAACTC	
LpRR6F	GCATCCTCCGCAGCTCCAAGT	KU136273
LpRR6R	CGGGCATCCAGTAGTCGGTGAT	
***LpRR10*F**	CCAACCAGCACCCATTCTCAGTC	KU136275
***LpRR10*R**	GCCGCCAGTGATACACCATTTGA	
***LpRR12a*F**	GCAGGATTCTAGTATATCCCAGCAGTGT	KU136276
***LpRR12a*R**	TGCCAGAAGAACGAGTTCCACATTTG	
***LpRR12b*F**	GTTCCACAAGCGAAGATTGATTTCCTC	KU136277
***LpRR12b*R**	AAGCCCCGAGCGAGTAGAAGTC	

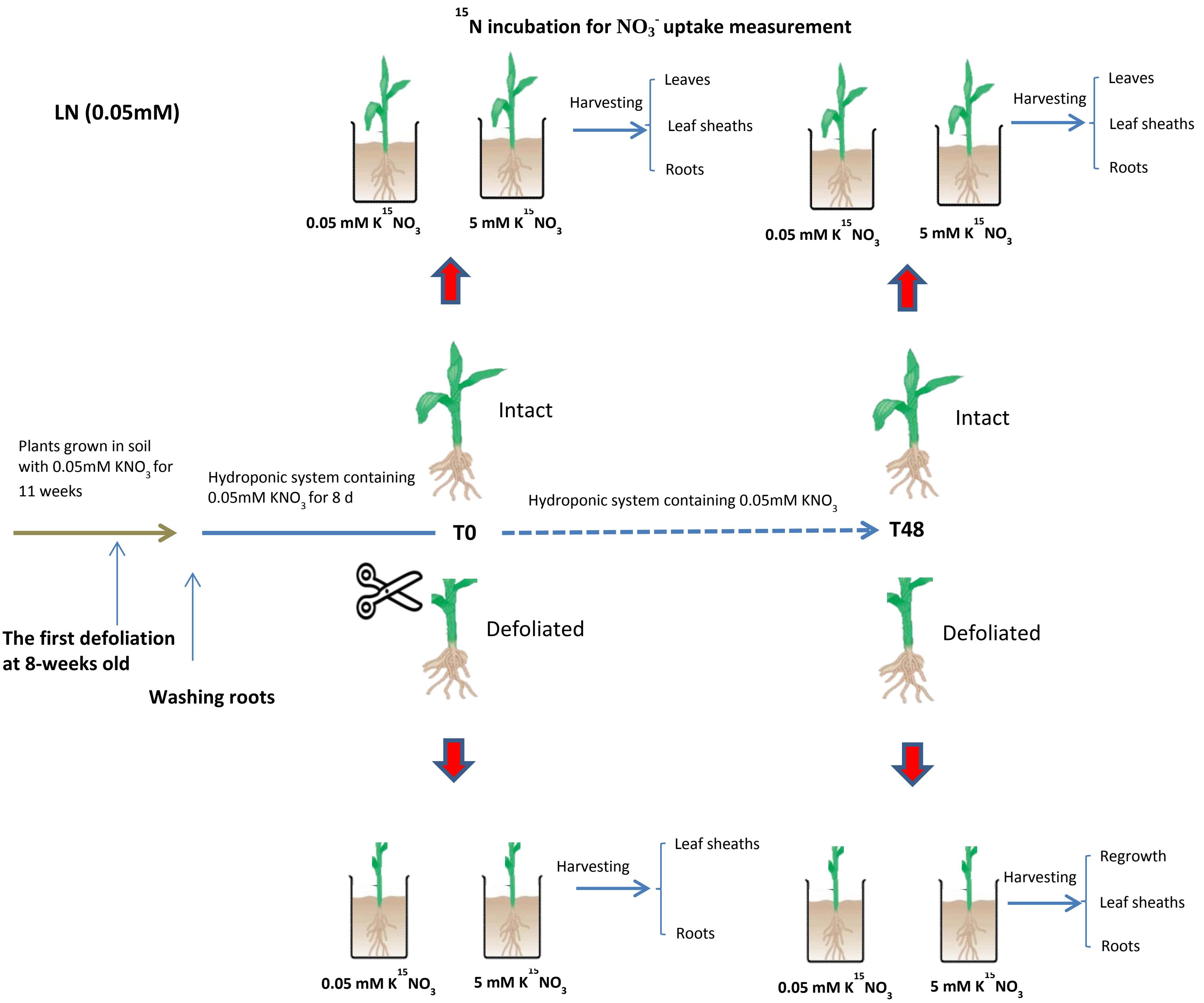
15N incubation for NO3- uptake measurement
LN (0.05mM)
Harvesting
Leaves
Leaf sheaths
Roots
0.05 mM K15NO3
5 mM K15NO3
Intact
Plants grown in soil with 0.05mM KNO3 for 11 weeks
Hydroponic system containing 0.05mM KNO3 for 8 d
Hydroponic system containing 0.05mM KNO3
T0
T48
Defoliated
The first defoliation at 8-weeks old
Washing roots
Regrowth

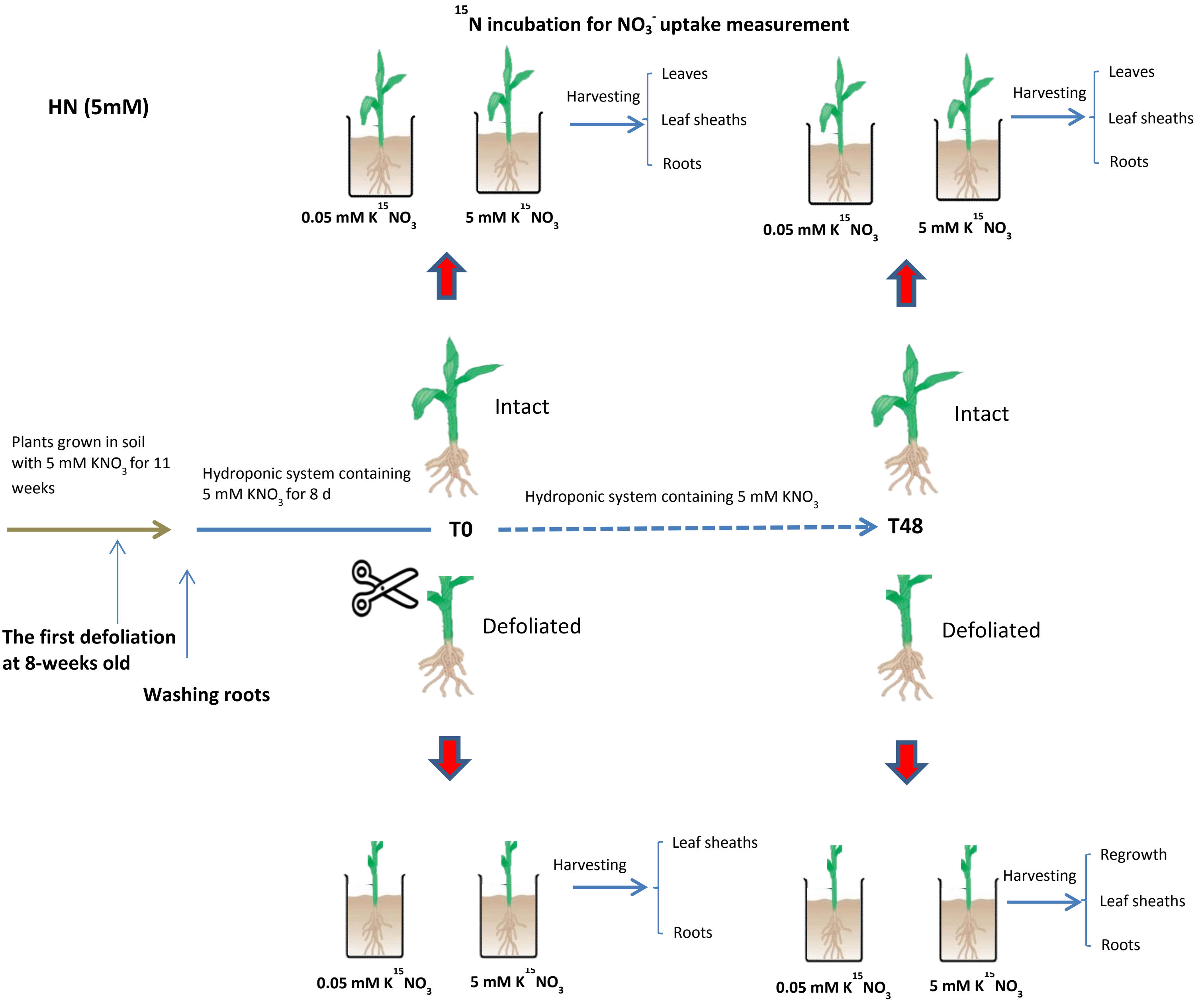

15N incubation for NO3- uptake measurement
HN (5mM)
Harvesting
Leaves
Leaf sheaths
Roots
0.05 mM K15NO3
5 mM K15NO3
Intact
Plants grown in soil with 5 mM KNO3 for 11 weeks
Hydroponic system containing 5 mM KNO3 for 8 d
Hydroponic system containing 5 mM KNO3
T0
T48
The first defoliation at 8-weeks old
Washing roots
Defoliated
Regrowth

Fig. S1. **Flow diagram of $K^{15}NO_3$ uptake measurement**

Seeds of *Lolium perenne* L. cv. Grasslands Nui were germinated in Eppendorf tubes filled with perlite (tube tips removed) and the tubes placed in unfertilised soil for 11 weeks. The basic N-free Hoagland medium was provided with 0.05 or 5 mM KNO_3 as a sole nitrogen source every week. Eight-week-old plants were defoliated at 4 cm above ground level. After 3-weeks regrowth, plant roots were washed and the plants transferred to a hydroponic system which contained basic nitrogen-free Hoagland medium supplemented with either 0.05 or 5 mM KNO_3. The plants remained in the tube to avoid transplant damage and the tubes were slotted into the hydroponic channels. Based on preliminary experiments, a one-week adaptation phase in liquid culture medium was required for plants to retain pre-transfer competence (as assessed by leaf gas exchange and stomatal conductance measurements). After the one-week adaptation plants were again defoliated. The time zero of the experiments is defined by the second defoliation of plants. After 0 and 48 h of regrowth, plants were gently blotted on tissue paper and then immediately rinsed with 0.1 mM $CaSO_4$ for 1 min to remove any adsorbed compounds on the root surface, followed by the exposure to basic nitrogen-free Hoagland medium supplemented with either 0.05 or 5 mM ^{15}N-labelled KNO_3 (atom % ^{15}N: 10%). During the uptake experiments the incubation solutions were aerated by an aquarium pump. At the end of the incubation period, roots were immediately rinsed with 0.1mM $CaSO_4$ for 1 min. Shoots and roots were separated, frozen in liquid nitrogen immediately and stored at -80°C.

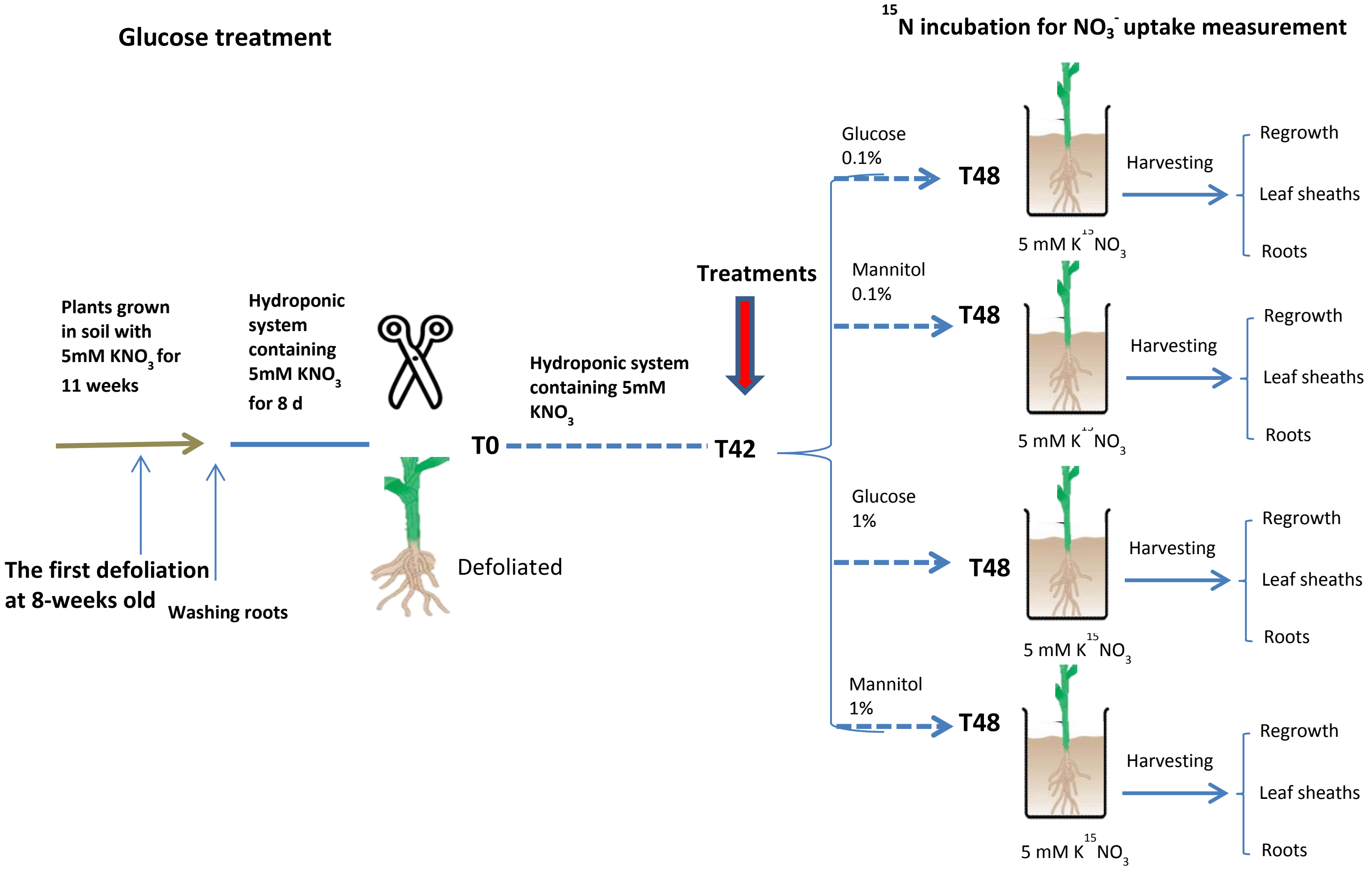

Fig. S2. **Flow diagram of K$^{15}NO_3$ uptake measurement in glucose rescue experiment**

A subset of HN plants as described in above Fig S1 was subjected to glucose treatment at 42 h after defoliation. 100 plants grown in solutions with 5 mM KNO_3 were then supplemented with either 0.1% or 1% (w/v) glucose (25 plants each) or in 0.1% or 1% (w/v) mannitol (25 plants each) as control. After 6 h, the K$^{15}NO_3$ uptake measurement was carried out by exposing roots to 5 mM ^{15}N-labelled KNO_3 (atom % ^{15}N: 10%) solutions as described above.

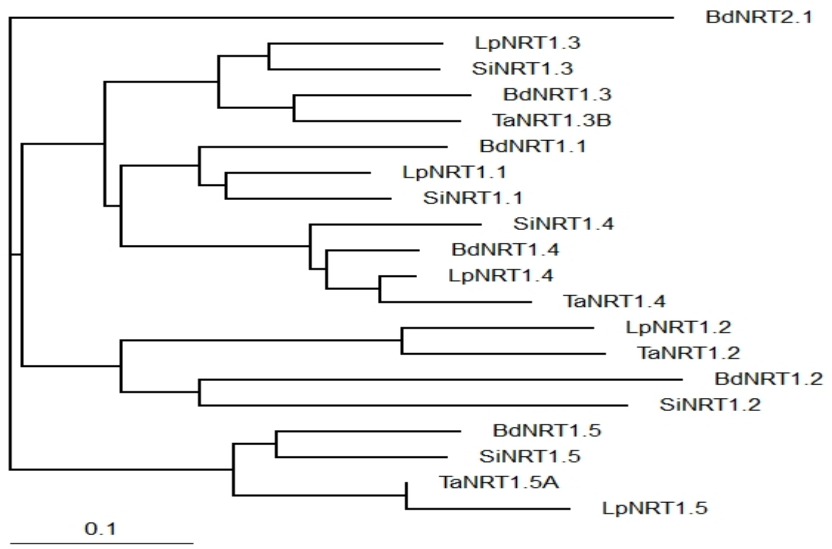
BdNRT2.1
LpNRT1.3
SiNRT1.3
BdNRT1.3
TaNRT1.3B
BdNRT1.1
LpNRT1.1
SiNRT1.1
SiNRT1.4
BdNRT1.4
LpNRT1.4
TaNRT1.4
LpNRT1.2
TaNRT1.2
BdNRT1.2
SiNRT1.2
BdNRT1.5
SiNRT1.5
TaNRT1.5A
LpNRT1.5
0.1

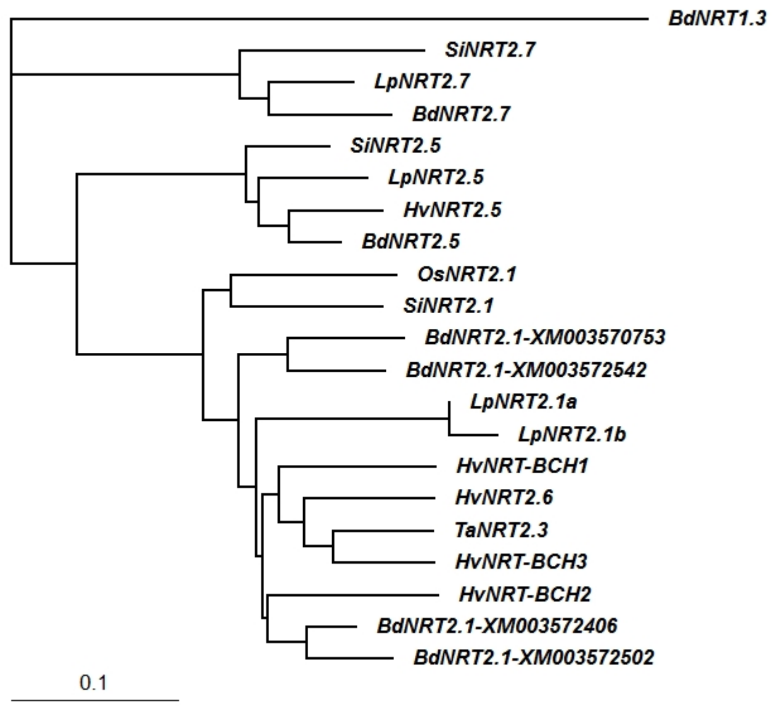
BdNRT1.3
SiNRT2.7
LpNRT2.7
BdNRT2.7
SiNRT2.5
LpNRT2.5
HvNRT2.5
BdNRT2.5
OsNRT2.1
SiNRT2.1
BdNRT2.1-XM003570753
BdNRT2.1-XM003572542
LpNRT2.1a
LpNRT2.1b
HvNRT-BCH1
HvNRT2.6
TaNRT2.3
HvNRT-BCH3
HvNRT-BCH2
BdNRT2.1-XM003572406
BdNRT2.1-XM003572502
0.1

.

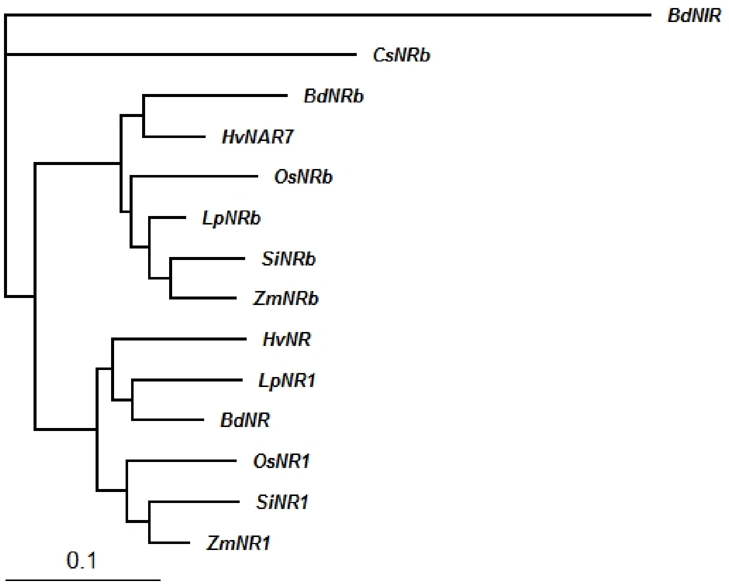

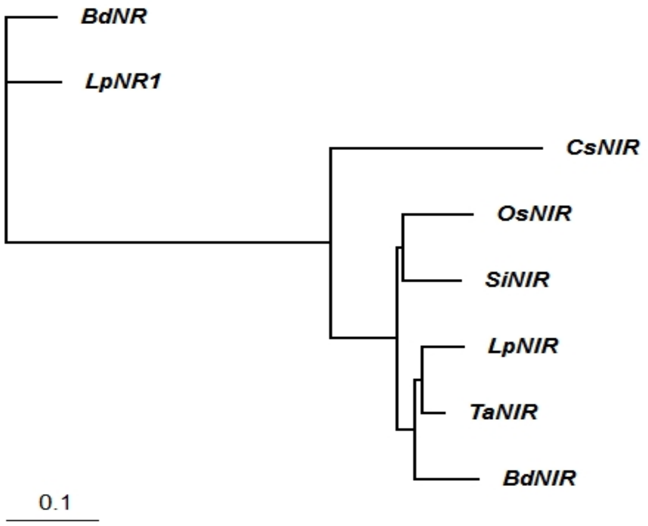

Fig. S3. **Phylogeny of *LpNRT*, *LpNR*, *LpNiR* gene families**

Neighbor-joining (NJ) phylogenetic trees of the newly identified sequences and their orthologues were created using ClustalX2 software with 1000 bootstrap replicates. The phylogenetic trees were visualised with TreeView X software. The trees were rooted with an out group sequence, *NRT2.1*, from *Brachypodium distachyon* Bd. *B. distachyon* Bd, *Lolium perenne* Lp, *Oryza brachyantha* Ob, *Oryza sativa* Os, *Seteria italica* Si, *Triticum aestivum* Ta, *Triticum urartu* Tu, *Zea mays* Zm.

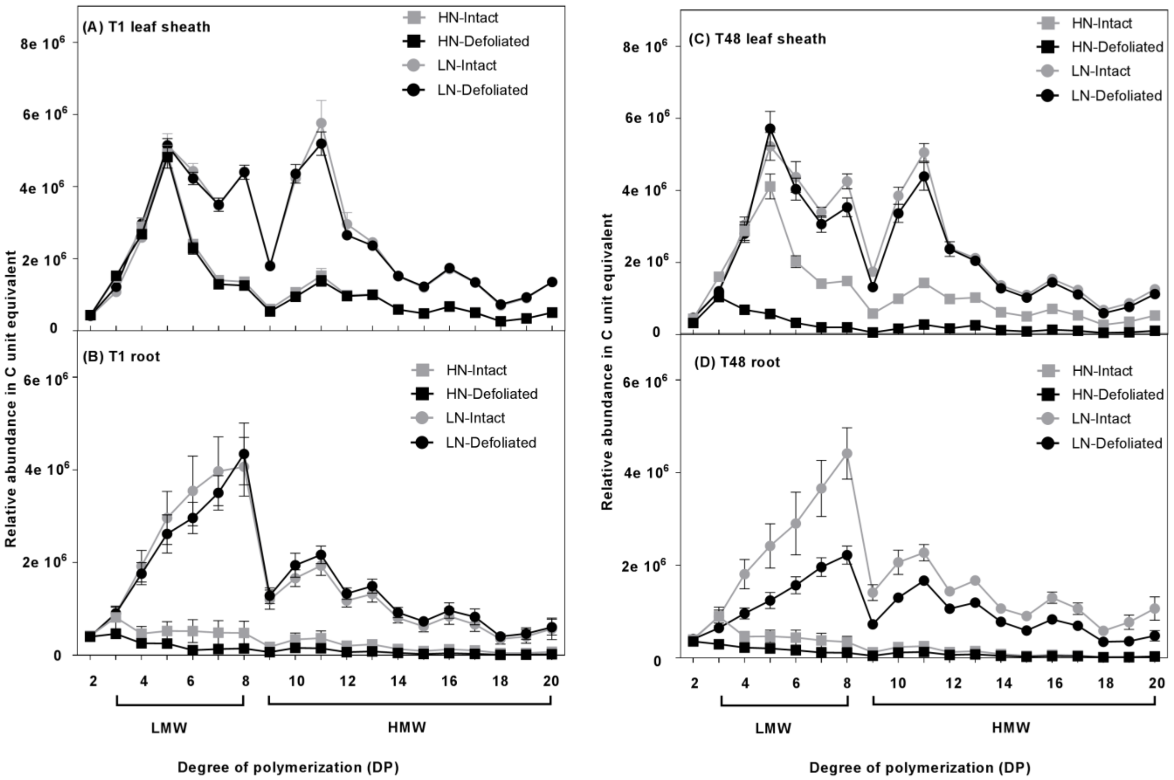

Fig. S4 Relative abundance in carbon (C) units of water-soluble carbohydrates (WSC). WSC were measured in plants grown under high and low nitrate supply, 1 h and 48 h after defoliation. Relative abundance in C units was calculated by multiplying peak intensity by degree of polymerization (DP). WSC profile in (A) leaf sheaths and (B) roots after 1 h; (C) leaf sheaths and (D) roots after 48 h. WSCs with degree of polymerization (DP) from three to eight are referred to here as low molecular weight (LMW) WSCs, and DP9 to DP20 are referred to as high molecular weight (HMW) WSCs. Values are means ± SEM (n=5 pools of five plants each).

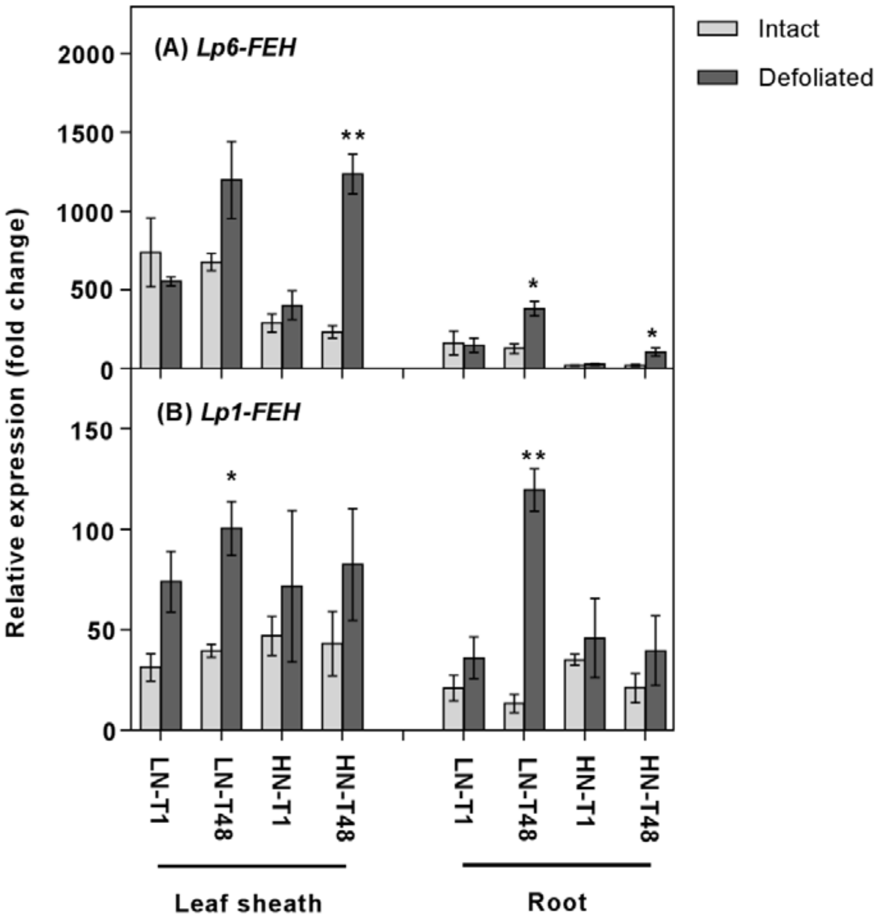

Fig. S5 Expression of putative *Lp6-FEH* and *Lp1-FEH* in plants grown under either 0.05 mM (LN) or 5 mM NO_3^- (HN) supply, 1 h and 48 h after defoliation. Each data point was normalized against reference genes *eEF-1α* and *GAPDH*. Values are means ± SEM (n=3 pools of five plants each). Means were tested for significance using a two-tailed t-test. *denotes significantly different means between intact plants (grey bars) and defoliated plants (black bars) (* *P<0.05*, ** *P<0.01*).

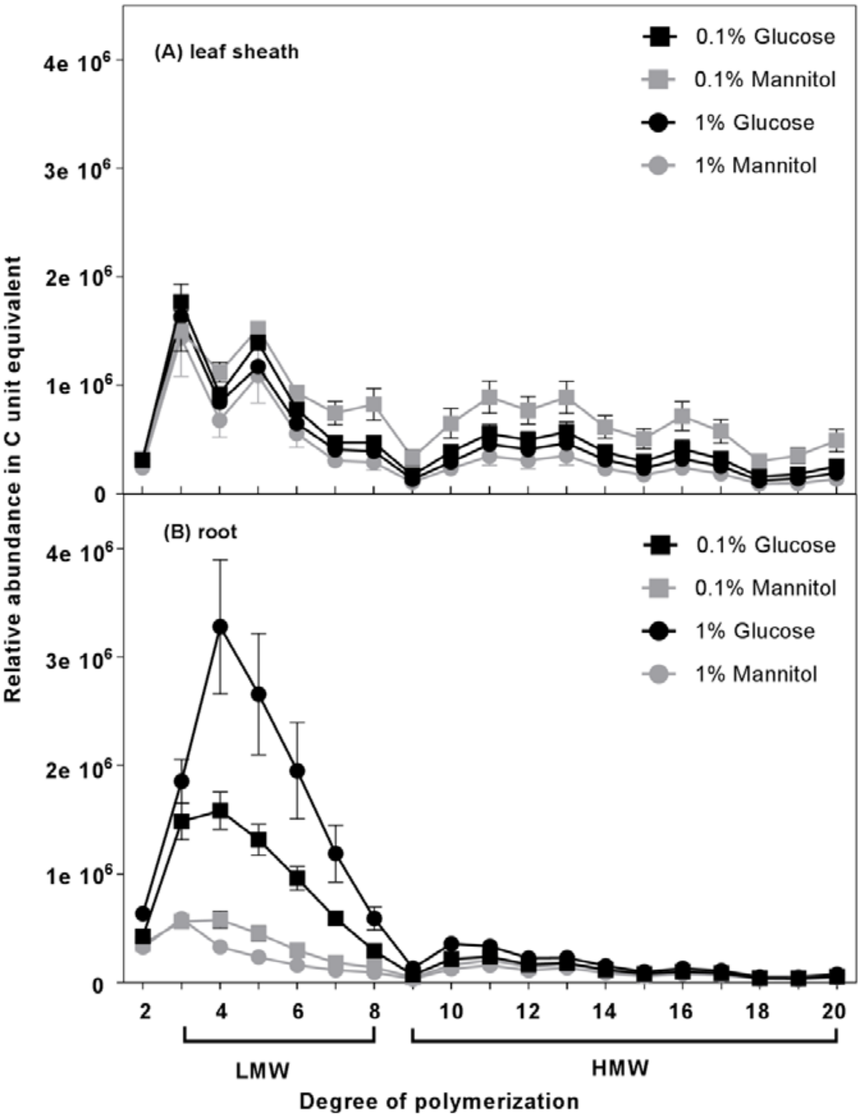

Fig. S6 . The impact of glucose on relative abundance in carbon (C) units of water-soluble carbohydrates (WSC). Plants were grown in HN conditions and supplied with 0.1% or 1% glucose 42 h after defoliation. After 6 h, WSC were measured and relative abundance in C units was calculated by multiplying peak intensity by degree of polymerization (DP). WSCs with degree of polymerization (DP) from three to eight are referred to here as low molecular weight (LMW) WSCs, and DP9 to DP20 are referred to as high molecular weight (HMW) WSCs. Values are means ± SEM (n=5 pools of five plants each).

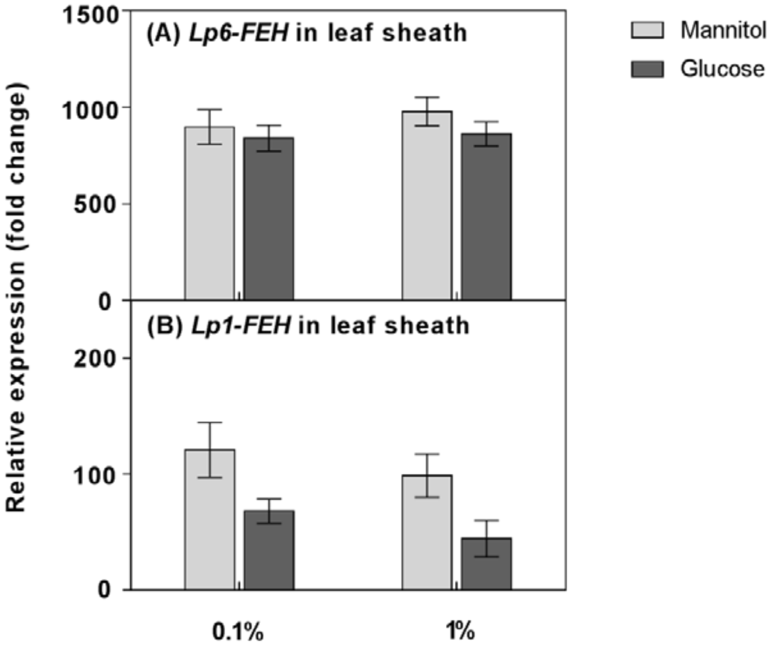

Fig. S7 ***Lp6-FEH*** **and** ***Lp1-FEH*** **expression in leaf sheaths with 6 h of supplemental glucose.** Plants were grown in HN conditions and supplied with 0.1% or 1% glucose 42 h after defoliation. Each data point was normalized against reference genes *eEF-1α* and *GAPDH*. Values are means ± SEM (n=3 pools of five plants each)

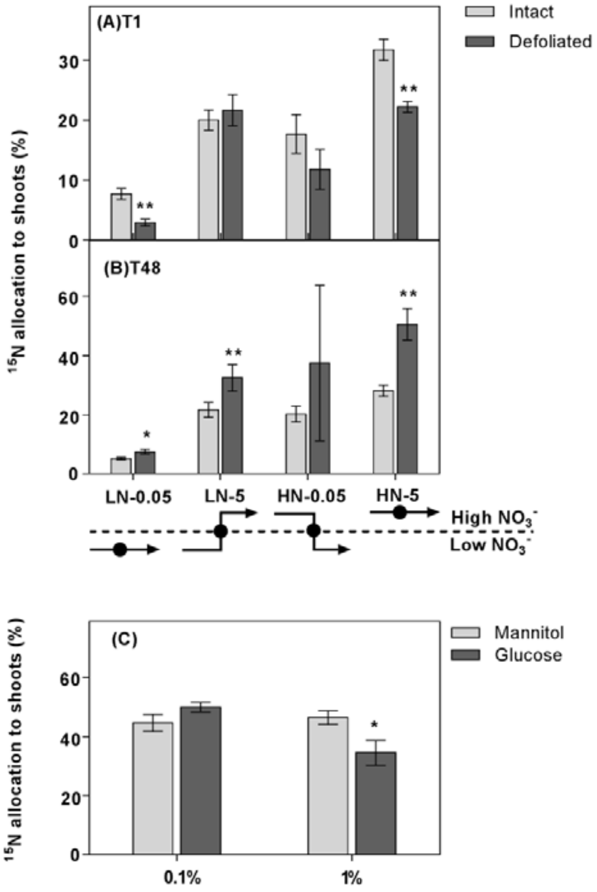

Fig. S8 The impact of defoliation or glucose addition on ^{15}N allocation to leaf sheaths. ^{15}N allocation to leaf sheaths was determined as the proportion of ^{15}N in leaf sheath to that in the total plant (roots and leaf sheaths). Values are means ± SEM (n=5 pools of five plants each). Means were tested for significance using a two-tailed t-test. *denotes significantly different means between intact plants (grey bars) and defoliated plants (black bars), or between mannitol treatment (grey bars) and glucose treatment (black bars) (* $P<0.05$, ** $P<0.01$).

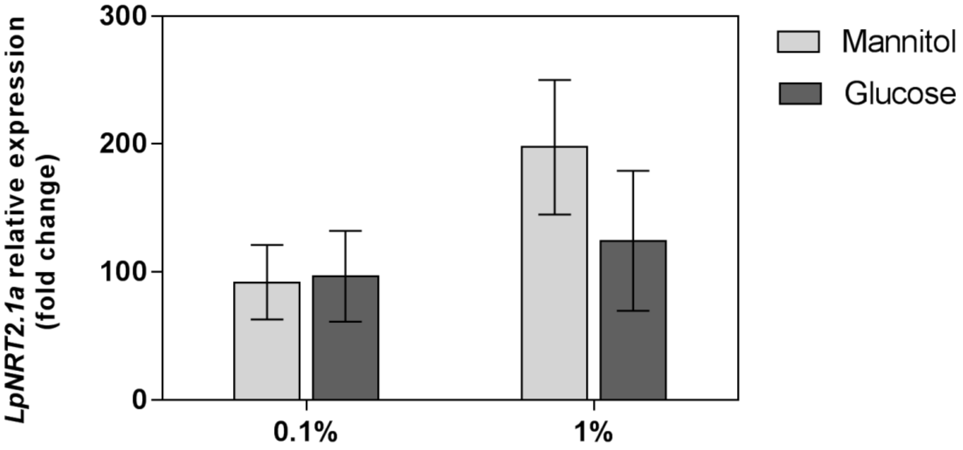

Fig. S9 Effects of 0.1% and 1% glucose addition on putative *LpNRT2.1a* gene expression in roots grown in 5 mM NO_3^-, 48 h after defoliation. Each data point is normalized against reference genes *eEF-1α* and *GAPDH*. Values are means ± SEM (n=3 pools of five plants each).

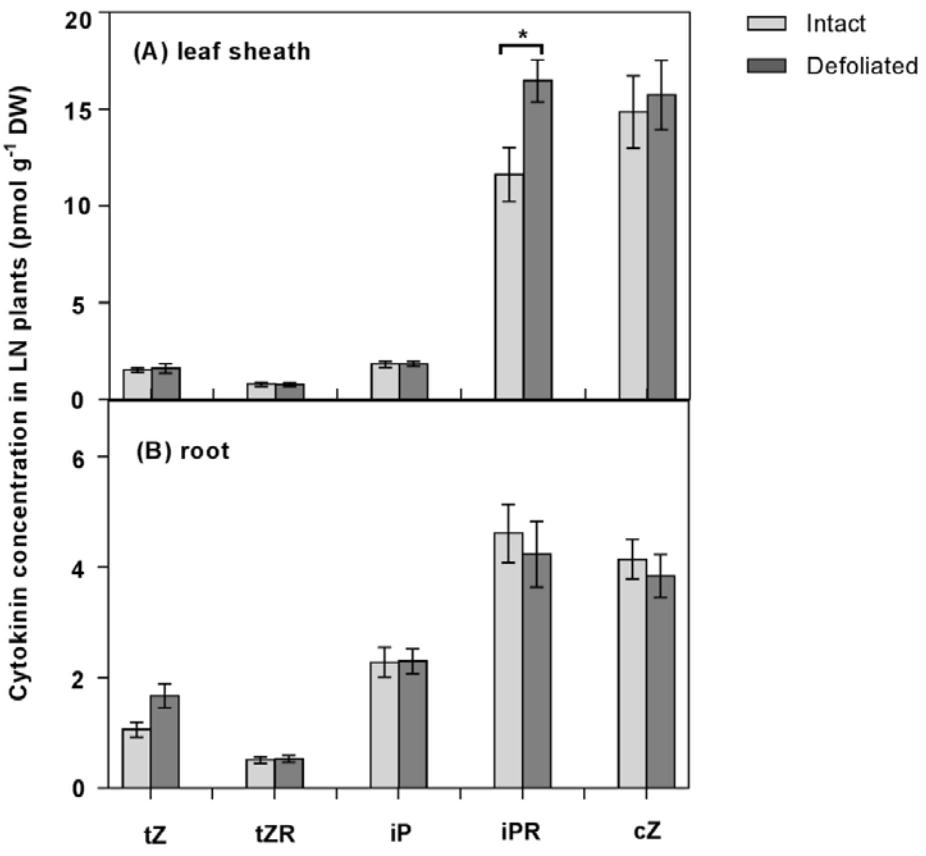

Fig. S10 Cytokinin concentrations in LN roots and leaf sheaths 48 h after defoliation. Values are means ± SEM (n=5 pools of five plants each).*denotes significantly different means between intact plants and defoliated plants. Means were tested for significance using a two-tailed t-test (* *P<0.05*).

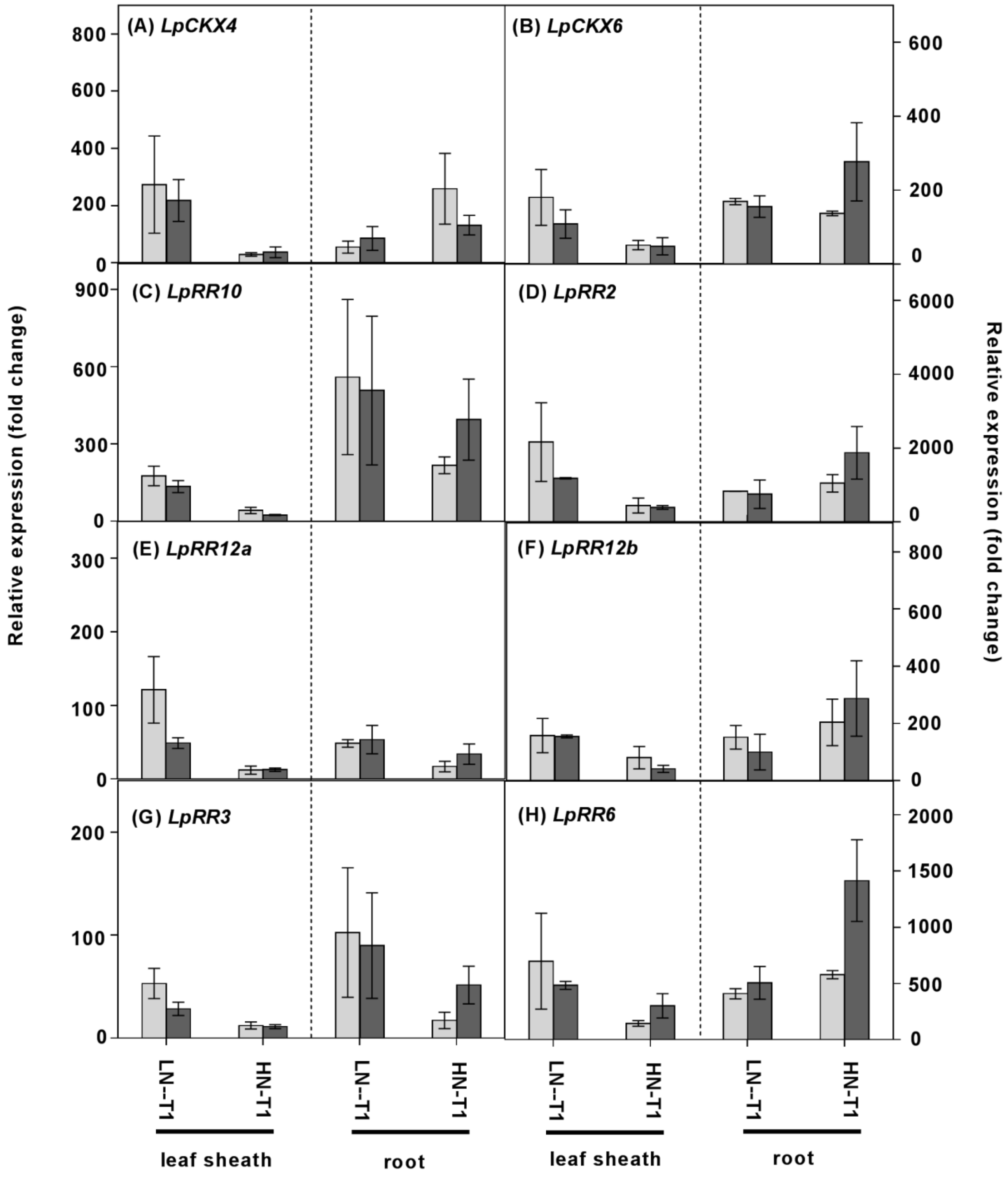

Fig. S11 The impact of 1-h defoliation on putative *LpCKX* and *LpRR* gene expression. Plants were grown at either 0.05 mM (LN) or 5 mM (HN) NO_3^- , and then defoliated (or left intact). Each data point was normalized against reference genes *eEF-1α* and *GAPDH*. Values are means ± SEM (n=3 pools of five plants each). Means were tested for significance using a two-tailed t-test.

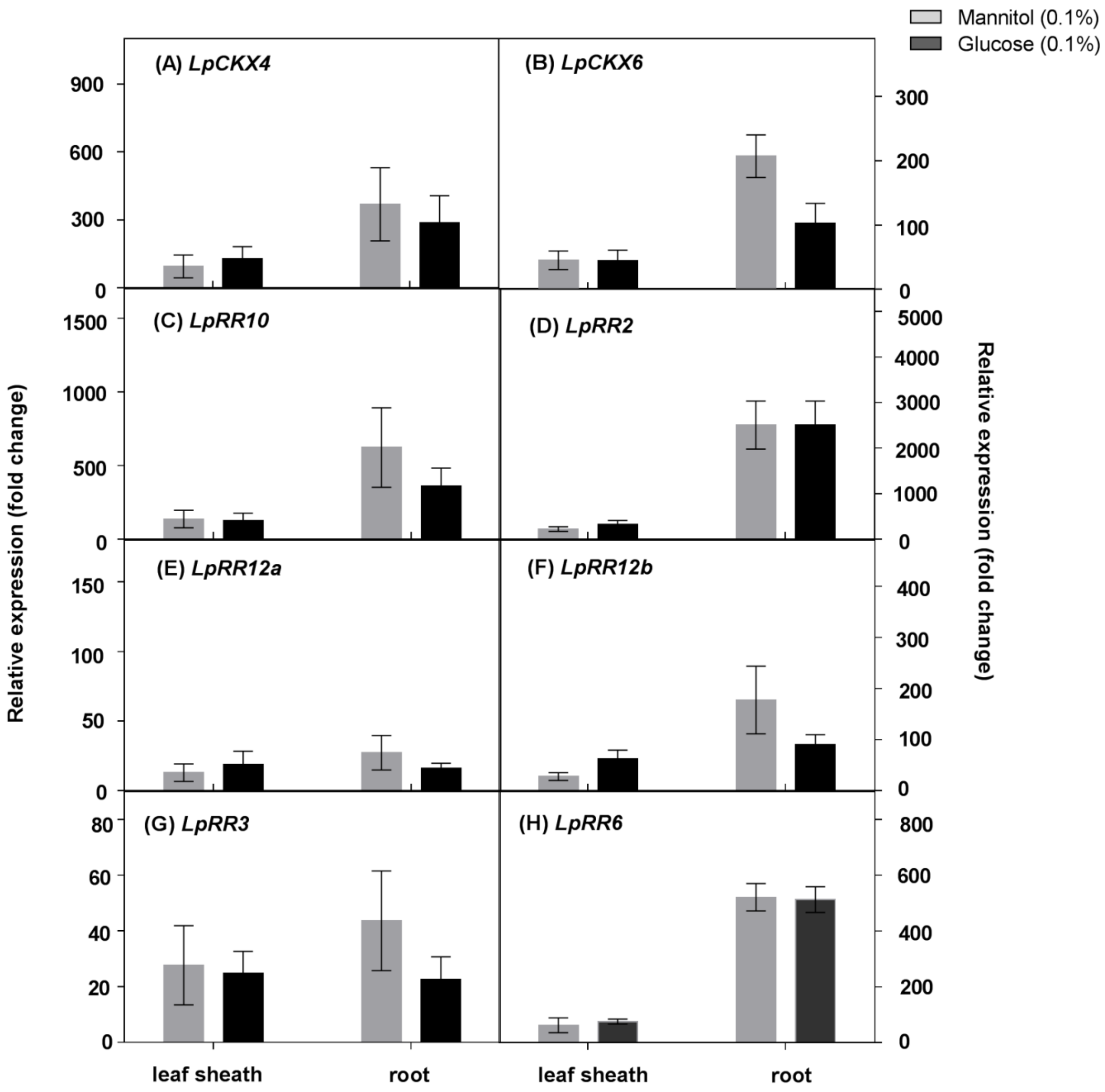

Fig. S12 Effects of 0.1% glucose addition on putative *LpCKX* and *LpRR* gene expression in plants grown in 5 mM NO_3^-, 48 h after defoliation. Each data point was normalized against reference genes *eEF-1α* and *GAPDH*. Values are means ± SEM (n=3 pools of five plants each). Means were tested for significance using a two-tailed t-test.
